# ﻿Two new hypogean species of the genus *Triplophysa* (Osteichthyes, Cypriniformes, Nemacheilidae) from Guizhou Province, Southwest China, with underestimated diversity

**DOI:** 10.3897/zookeys.1214.122439

**Published:** 2024-10-09

**Authors:** Chang-Ting Lan, Li Wu, Tao Luo, Ye-Wei Liu, Jia-Jun Zhou, Jing Yu, Xin-Rui Zhao, Ning Xiao, Jiang Zhou

**Affiliations:** 1 School of Life Sciences, Guizhou Normal University, Guiyang 550025, Guizhou, China; 2 School of Karst Science, Guizhou Normal University, Guiyang 550001, Guizhou, China; 3 Guangxi Key Laboratory for Forest Ecology and Conservation, College of Forestry, Guangxi University, Nanning 530004, Guangxi, China; 4 Zhejiang Forest Resource Monitoring Center, Hangzhou 310020, Zhejiang, China; 5 Zhejiang Forestry Survey Planning and Design Company Limited, Hangzhou 310020, Zhejiang, China; 6 Guiyang Healthcare Vocational University, Guiyang 550081, Guizhou, China

**Keywords:** Mitochondrial DNA, morphology, new species, nuclear gene, *Triplophysa*

## Abstract

Two new species of the genus *Triplophysa* from southwestern Guizhou Province, China, are described. These are *Triplophysaziyunensis* Wu, Luo, Xiao & Zhou, **sp. nov.** and *Triplophysayaluwang* Lan, Liu, Zhou & Zhou, **sp. nov.** from Maoying Town, Ziyun County, Guizhou Province, China. *Triplophysaziyunensis* Wu, Luo, Xiao & Zhou, **sp. nov.** is distinguished from other hypogean species of the genus *Triplophysa* by having a combination of the following characteristics: body naked, scaleless, pigmented markings on surface of body, except ventral; eyes reduced, diameter 2.4–4.9% of head length; pelvic-fin tip extending to anus; tip of pectoral fin not reaching pelvic fin origin; anterior and posterior nostrils closely set, with anterior nostril elongated to a barbel-like tip; tip of outrostral barbel extending backward, not reaching anterior margin of the eye; lateral line complete; posterior chamber of air bladder degenerated; and fin differences. *Triplophysayaluwang* Lan, Liu, Zhou & Zhou, **sp. nov.** is distinguished from other hypogean species of the genus *Triplophysa* by having a combination of the following characteristics: body naked, scaleless, with irregular pale and dark brownish brown markings, except ventrally; eyes reduced, diameter 4.6–6.1% of head length; pelvic-fin tip reaching anus; tip of pectoral fin not reaching to pelvic fin origin; anterior and posterior nostrils closely set, with anterior nostril elongated to a barbel-like tip; tip of outrostral barbel extending backward, not reaching to anterior margin of the eye; lateral line complete; posterior chamber of air bladder degenerated; and fin differences. Mitochondrial Cyt *b* revealed relatively small genetic differences, 1.4–2.0%, between the two new species and close relatives. Nuclear gene RAG1 indicated that the two new species possessed unique haplotypes with multiple linking mutations. This study emphasizes the importance of utilizing nuclear genes to identify new species in rapidly speciation cave species, with small genetic differences due to mitochondrial introgression occurring interspecies.

## ﻿Introduction

The high-plateau loach fish genus *Triplophysa* Rendahl, 1933 comprises more than 184 recognized species of small loaches that are distributed on the Qinghai-Xizang Plateau and nearby regions (Luo et al. 2023; Fricke et al. 2024). Morphological characteristics that distinguish *Triplophysa* from other genera in the Nemacheilidae include closely set anterior and posterior nostrils and a posterior wall of the bony capsule of the swim bladder. Males have tubercle-bearing, elevated skin on both sides of the head and a thickened tuberculated pad or agglomerations on the dorsal surfaces of the broadened and widened pectoral-fin rays (Zhu 1989; Zheng et al. 2009; Prokofiev 2010; He et al. 2012; Ren et al. 2012; Yang et al. 2012; Wu et al. 2018a; Chen and Peng 2019; Deng et al. 2022). *Triplophysa* species are distributed from the Qinghai-Xizang Plateau at an average elevation of 4000 m to the Yunnan-Guizhou Plateau at an average elevation of 1000–2000 m (Zhu 1989; Luo et al. 2023). Their habitats include lakes, rivers, and caves (Zhu 1989; Lan et al. 2013), on the basis of which Triplophysa can be divided into two life groups: An epigean group and a hypogean or cave-dwelling group (Liu et al. 2022; Lu et al. 2022). The hypogean group is mainly distributed in underground rivers in southwest China, including Guizhou, Chongqing, Guangxi, Yunnan, and Hunan provinces (Luo et al. 2023). This group can be further subdivided into two morphological types, the stygobionts, and stygophiles (Zhao et al. 2011; Ma et al. 2019), based on the level of adaptation to the cave environment (Zhao et al. 2011; Lu et al. 2022). There are 106 species of *Triplophysa* and 39 hypogean species are distributed in Chongqing, Guangxi, Guizhou, Yunnan, and Hunan provinces in China (Table 1) (Lan et al. 2013; Zhang et al. 2020; Chen et al. 2021; Liu et al. 2022; Lu et al. 2022; Luo et al. 2023).

**Table 1.** A list of 39 species of hypogean fishes of the genus *Triplophysa* distributed in the Southwest China.

ID	Species	Province	Main drainage	Tributary	Reference
1	*T.aluensis* Li & Zhu, 2000	Yunnan	Pearl River	Nanpanjiang River	Li and Zhu 2000
2	*T.anshuiensis* Wu, Wei, Lan & Du, 2018	Guangxi	Pearl River	Hongshui River	Wu et al. 2018a
3	*T.anlongensis* Lan, Song, Luo, Zhao, Xiao & Zhou, 2023	Guizhou	Pearl River	Nanpanjiang River	Luo et al. 2023
4	*T.baotianensis* Li, Liu & Li, 2018	Guizhou	Pearl River	Nanpanjiang River	Li et al. 2018
5	*T.cehengensis* Luo, Mao, Zhao, Xiao & Zhou, 2023	Guizhou	Pearl River	Beipanjiang River	Luo et al. 2023
6	*T.erythraea* Liu & Huang, 2019	Hunan	Yangtze River	Yuanjiang River	Huang et al. 2019
7	*T.fengshanensis* Lan, 2013	Guangxi	Pearl River	Hongshui River	Lan et al. 2013
8	*T.flavicorpus* Yang, Chen & Lan, 2004	Guangxi	Pearl River	Hongshui River	Yang et al. 2004
9	*T.gejiuensis* (Chu & Chen, 1979)	Yunnan	Pearl River	Nanpanjiang River	Chu and Chen 1979
10	*T.guizhouensis* Wu, He & Yang, 2018	Guizhou	Pearl River	Hongshui River	Wu et al. 2018b
11	*T.huapingensis* Zheng, Yang & Che, 2012	Guangxi	Pearl River	Hongshui River	Zheng et al. 2012
12	*T.langpingensis* Yang, 2013	Guangxi	Pearl River	Hongshui River	Lan et al. 2013
13	*T.longipectoralis* Zheng, Du, Chen & Yang, 2009	Guangxi	Pearl River	Liujiang River	Zheng et al. 2009
14	*T.longliensis* Ren, Yang & Chen, 2012	Guizhou	Pearl River	Hongshui River	Ren et al. 2012
15	*T.luochengensis* Li, Lan, Chen & Du, 2017	Guangxi	Pearl River	Hongshui River	Li et al. 2017a
16	*T.macrocephala* Yang, Wu & Yang, 2012	Guangxi	Pearl River	Liujiang River	Yang et al. 2012
17	*T.nandanensis*Lan, Yang & Chen, 1995	Guangxi	Pearl River	Hongshui River	Lan et al. 1995
18	*T.nanpanjiangensis*Zhu & Cao, 1988	Yunnan	Pearl River	Nanpanjiang River	Zhu and Cao 1988
19	*T.nasobarbatula* Wang & Li, 2001	Guizhou	Pearl River	Liujiang River	Wang and Li 2001
20	*T.panzhouensis* Yu, Luo, Lan, Xiao & Zhou, 2023	Guizhou	Pearl River	Nanpanjiang River	Luo et al. 2023
21	*T.posterodorsalus* (Li, Ran & Chen, 2006)	Guangxi	Pearl River	Liujiang River	Ran et al. 2006
22	*T.qingzhenensis* Liu, Zen, & Gong, 2022	Guizhou	Yangtze River	Wujiang River	Liu et al. 2022
23	*T.qini* Deng, Wang & Zhang, 2022	Chongqing	Yangtze River	Yuanjiang River	Deng et al. 2022
24	*T.qiubeiensis* Li & Yang, 2008	Yunnan	Pearl River	Nanpanjiang River	Li et al. 2008
25	*T.rongduensis* Mao, Zhao, Yu, Xiao & Zhou,2023	Guizhou	Pearl River	Beipanjiang River	Luo et al. 2023
26	*T.rosa* Chen & Yang, 2005	Chongqing	Yangtze River	Wujiang River	Chen and Yang 2005
27	*T.sanduensis*Chen & Peng, 2019	Guizhou	Pearl River	Duliujiang River	Chen and Peng 2019
28	*T.shilinensis* Chen,Yang & Xu, 1992	Yunnan	Pearl River	Nanpangjiang River	Chen et al. 1992
29	*T.tianeensis* Chen, Cui & Yang, 2004	Guangxi	Pearl River	Hongshui River	Chen et al. 2004
30	*T.tianlinensis* Li, Li, Lan & Du, 2017	Yunnan	Pearl River	Hongshui River	Li et al. 2017b
31	*T.tianxingensis* Yang, Li & Chen, 2016	Yunnan	Pearl River	Nanpangjiang River	Yang et al. 2016
32	*T.wudangensis* Liu, Zen & Gong, 2022	Guizhou	Yangtze River	Wujiang River	Liu et al. 2022
33	*T.wulongensis* Chen, Sheraliev, Shu & Peng, 2021	Chongqing	Yangtze River	Wujiang River	Chen et al. 2021
34	*T.xiangshuingensis* Li, 2004	Yunnan	Pearl River	Nanpanjiang River	Li 2004
35	*T.xiangxiensis* Yang, Yuan & Liao, 1986	Hunan	Yangtze River	Yuanjiang River	Yang et al. 1986
36	*T.xichouensis* Liu, Pan, Yang & Chen, 2017	Yunnan	Red River	Red River	Liu et al. 2017
37	*T.xuanweiensis* Lu, Li, Mao & Zhao, 2022	Yunnan	Pearl River	Beipanjiang River	Lu et al. 2022
38	*T.yunnanensis* Yang, 1990	Yunnan	Pearl River	Nanpanjiang River	Chu and Chen 1990
39	*T.zhenfengensis* Wang & Li, 2001	Guizhou	Pearl River	Beipanjiang River	Wang and Li 2001

**Figure 1.**
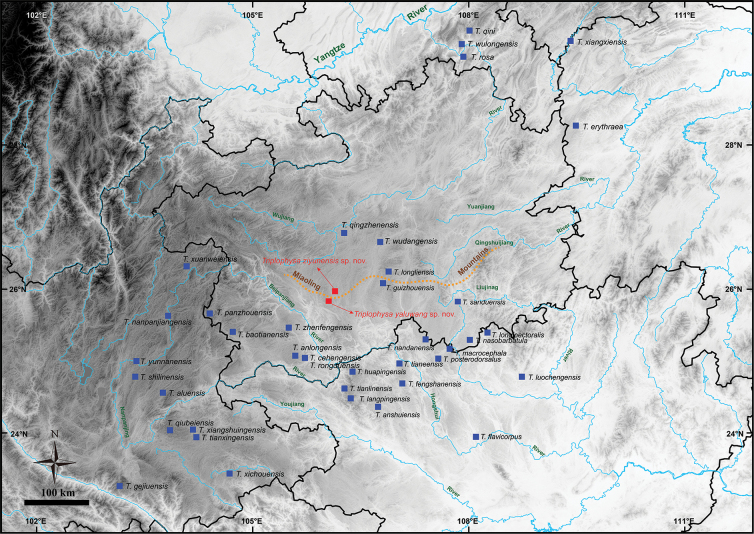
Sample collection localities and distributions of the two new species and 39 hypogean species of the genus *Triplophysa* in southern China. The base maps are from the Standard Map Service website (http://bzdt.ch.mnr.gov.cn/index.html).

Guizhou Province is the region where the two major rivers of Asia, the Pearl River, and the Yangtze River, are separated (Fig. 1). The subtropical monsoon climate and paleogeology have shaped the karst landscapes and rich cave resources. This ecological background has enabled the formation of a diverse subterranean biota (Li et al. 2022; Wen et al. 2022). A total of 12 species occur in this region, eight of which have been described during the last ten years. These are *Triplophysaanlongensis* Lan, Song, Luo, Zhao, Xiao & Zhou, 2023, *T.baotianensis* Li, Liu & Li, 2018, *T.guizhouensis* Wu, He & Yang, 2018, *T.cehengensis* Luo, Mao, Zhao, Xiao & Zhou, 2023, *T.longliensis* Ren, Yang & Chen, 2012, *T.nasobarbatula* Wang & Li, 2001, *T.panzhouensis* Yu, Luo, Lan, Xiao & Zhou, 2023, *T.qingzhenensis* Liu, Zen & Gong, 2022, *T.rongduensis* Mao, Zhao, Yu, Xiao & Zhou, 2023, *T.sanduensis* Chen & Peng, 2019, *T.wudangensis* Liu, Zen & Gong, 2022, and *T.zhenfengensis* Wang & Li, 2001 (Wang and Li 2001; Ren et al. 2012; Li et al. 2018; Wu et al. 2018a; Chen and Peng 2019; Liu et al. 2022; Luo et al. 2023). Body pigmentation and pigmented markings were present in 11 of the 12 species, except for *T.cehengensis*, and the key distinguishing characteristics of these species are shown in Table 2. Four new species of *Triplophysa* were recently described in Guizhou (Luo et al. 2023), thus suggesting that additional undescribed species may exist in this region.

**Table 2.** Comparison of the diagnostic features of the two new species described here with those selected for the 39 recognized hypogean species of the genus *Triplophysa*. Modified from Wu et al. (2018b), Liu et al. (2022), and Luo et al. (2023). “–” indicates uncertainty.

ID	Species	Body pigmentation	Eyes	Eye diameter (% HL)	Interorbital width (% HL)	Dorsal fin distal margin	Secondary sex characteristics	Scales	Lateral line	Posterior chamber of air bladder	Dorsal fin rays	Analfin rays	Pectoral fin rays	Pelvic fin rays	Caudalfin rays	Tip of pelvic fin reaching anus	Anterior nostril barbel-like	Vertebrae
1	*T.yaluwang* sp. nov.	Entire body	Reduced	4.6–6.1	24.3–26.0	Emarginated	Absent	Absent	Complete	Degenerated	iii, 7	iii, 5	i, 9	i, 5–6	14	Yes	Yes	4 + 36
2	*T.ziyunensis* sp. nov.	Entire body	Reduced	2.4–4.9	22.3–26.2	Truncated	Absent	Absent	Complete	Degenerated	iii, 8	iii, 5	i, 10	i, 6	16	Yes	Yes	4 + 35
3	*T.aluensis*	Absent	Reduced	5.6	22.2	Truncated	–	Absent	Complete	Degenerated	iii, 7	iii, 5	i, 9	i, 6	13	No	Yes	–
4	*T.anlongensis*	Entire body	Normal	5.1–9.3	32.1–35.6	Truncated	Absent	Absent	Complete	Degenerated	iii, 8	iii, 5	i, 11	ii, 8	16	No	Yes	4 + 37
5	*T.anshuiensis*	Dorsal	Absent	Absent	–	Truncated	Absent	Absent	Complete	Developed	iv, 7–8	ii, 6	i, 10	i, 6	14	Yes	Yes	–
6	*T.baotianensis*	Entire body	Normal	14.0–15.0	3.40–4.57	Truncated	–	Absent	Complete	Degenerated	iii, 6–7	ii, 4–5	i, 9	i, 5	11–13	No	Yes	–
7	*T.cehengensis*	Absent	Reduced	1.5–2.2	27.2–36.5	Emarginated	Absent	Absent	Complete	Developed	iv, 9	iii, 5	i, 10	ii, 8	16	Yes	Yes	4 + 35
8	*T.erythraea*	Absent	Absent	Absent	–	Truncated	Absent	Absent	Complete	Developed	ii, 8	i, 6	ii, 10	ii, 5	17	Yes	No	–
9	*T.fengshanensis*	Absent	Absent	Absent	–	Truncated	–	Absent	Complete	–	ii, 8	ii, 6	i, 8–10	i, 6–7	16	No	Yes	–
10	*T.flavicorpus*	Entire body	Normal	5.1–6.8	3.1–5.2	Emarginated	–	Present	Complete	Degenerated	iii, 10	iii, 6–7	i, 11	i, 6–7	16	Yes	No	4 + 34
11	*T.gejiuensis*	Absent	Absent	Absent	–	Truncated	–	Absent	Complete	Developed	iii, 7–8	iii, 4–6	i, 10	i, 5	14–15	Yes	Yes	–
12	*T.guizhouensis*	Entire body	Normal	9.4–12.1	20.3–24.3	Truncated	Absent	Present	Complete	Developed	iii, 8	iii, 6	i, 8–9	i, 6	14	No	Yes	–
13	*T.huapingensis*	Entire body	Normal	10.4–14.3	27.6–30.8	Truncated	Present	Present	Complete	Degenerated	iii, 8–9	iii, 5	i, 9–10	i, 5–6	16	No	No	–
14	*T.langpingensis*	Absent	Reduced	2.7–5.9	30.6–34.5	Truncated	–	Absent	Incomplete	–	iii, 7–8	iii, 5–6	i, 10–11	i, 6	14	Yes	Yes	–
15	*T.longipectoralis*	Entire body	Normal	11.8–16.4	21.2–25.3	Emarginated	Present	Present	Complete	Degenerated	iii, 8	iii, 5–6	i, 9–10	i, 6	14–15	Yes	Yes	4 + 35
16	*T.longliensis*	Entire body	Normal	9.5–11.5	31.4–37.5	Emarginated	Present	Absent	Complete	Developed	iii, 8	iii, 5	i, 10	i, 6	15–16	Yes	Yes	4 + 38
17	*T.luochengensis*	Entire body	Reduced	7.5–8.6	18.4–21.3	Truncated	Present	Present	Complete	Degenerated	iii, 8	ii, 6	i, 10	i, 6	16–17	No	Yes	4 + 33–34
18	*T.macrocephala*	Entire body	Reduced	3.6–8.0	22.9–25.8	Truncated	Present	Absent	Complete	Degenerated	iii, 7–9	iii, 5–6	i, 9–11	i, 6	15–17	Yes	Yes	–
19	*T.nandanensis*	Entire body	Normal	11.1–21.3	24.4–27.8	Emarginated	–	Present	Complete	Degenerated	iv, 8	iv, 5	i, 9–10	i, 6	14–16	No	Yes	4 + 36
20	*T.nanpanjiangensis*	Entire body	Normal	12.0–16.5	30.3–34.5	Truncated	Present	Absent	Complete	Degenerated	iii, 7–8	ii, 5	i, 9–10	i, 6	16	No	Yes	4 + 38
21	*T.nasobarbatula*	Entire body	Normal	9.1–13.3	27.0–33.3	Truncated	–	Present	Complete	Degenerated	iii, 8	iii, 5	i, 9	i, 6	15	Yes	Yes	4 + 36
22	*T.panzhouensis*	Entire body	Normal	7.0–11.0	22.1–31.3	Truncated	Absent	Absent	Complete	Degenerated	iv, 7–8	iii, 5	i, 11	ii, 7	16	No	Yes	4 + 35
23	*T.posterodorsalus*	Absent	Absent	Absent	–	Truncated	–	Absent	Complete	–	iii, 6	ii, 4	i, 13	i, 5	15	No	Yes	–
24	*T.qingzhenensis*	Entire body	Reduced	2.1–4.4	25.1–30.4	Truncated	Absent	Absent	Complete	Degenerated	iii, 7–8	iii, 5	i, 8–9	i, 5	14	No	Yes	4 + 36
25	*T.qini*	Absent	Absent	Absent	–	Emarginated	Present	Absent	Complete	–	ii, 8	ii, 5	–	–	14–16	Yes	No	4 + 34–35
26	*T.qiubeiensis*	Absent	Absent	Absent	–	Emarginated	–	Absent	Complete	Degenerated	iii, 7	iii, 5	i, 7–9	i, 5	14–15	Yes	No	4 + 35
27	*T.rongduensis*	Entire body	Normal	7.2–14.7	24.1–28.6	Truncated	Absent	Absent	Complete	Degenerated	iv, 9	iii, 5	i, 10	ii, 7	16	No	Yes	4 + 39
28	*T.rosa*	Absent	Absent	Absent	–	Emarginated	Absent	Absent	Complete	–	iii, 9	iii, 6	i, 12	i, 7	14	Yes	Yes	–
29	*T.sanduensis*	Entire body	Normal	11.9–15.4	31.2–40.2	Emarginated	Present	Present	Complete	Degenerated	ii, 8–9	i, 5	i, 8–9	i, 5	17–18	No	Yes	4 + 37
30	*T.shilinensis*	Absent	Absent	Absent	–	Truncated	–	Absent	Complete	Degenerated	iii, 7	iii, 5	i, 8–10	i, 6	14	No	Yes	–
31	*T.tianeensis*	Entire body	Reduced	3.0–5.9	21.3–25.6	Truncated	Present	Absent	Complete	Degenerated	iii, 6–7	iii, 6	i, 8–9	i, 5–6	15–16	No	Yes	4 + 35
32	*T.tianlinensis*	Absent	Reduced	Absent	Absent	Truncated	Present	Absent	Complete	Degenerated	iv, 8–9	iii, 6	i, 10	i, 6	15–16	Yes	Yes	–
33	*T.tianxingensis*	Entire body	Normal	4.2–6.7	17.4–24.0	Truncated	Absent	Absent	Complete	Developed	iii, 8	ii, 5	i, 9	i, 5	16	No	No	4 + 38
34	*T.wudangensis*	Entire body	Reduced	5.1–6.5	33.1–35.8	Truncated	Absent	Absent	Complete	Degenerated	iii, 7	iii, 5	i, 8	i, 5	14	No	Yes	4 + 34
35	*T.wulongensis*	Entire body	Normal	11.1–19.1	38.5–43.1	Emarginated	–	Absent	Complete	Degenerated	ii, 8–9	i, 5–6	i, 8–9	i, 5–7	18	No	Yes	4 + 38–39
36	*T.xiangshuingensis*	Entire body	Normal	7.5	32.3	Emarginated	–	Absent	Complete	Degenerated	iii, 6	iii, 5	i, 9	i, 6	14	No	Yes	–
37	*T.xiangxiensis*	Absent	Absent	Absent	–	Emarginated	–	Absent	Complete	Developed	iii, 8	iii, 6	i, 11	i, 6	16	Yes	Yes	–
38	*T.xichouensis*	Entire body	Reduced	Absent	–	Truncated	Absent	Absent	Complete	Developed	iii, 8	ii, 6	i, 9–10	i, 5–6	16	Yes	Yes	4 + 36
39	*T.xuanweiensis*	Absent	Absent	Absent	–	Emarginated	–	Absent	Complete	Well developed	iii, 7–8	iii, 5	i, 10–12	i, 7–8	17–18	Yes	No	–
40	*T.yunnanensis*	Entire body	Normal	7.2–8.3	27.0–27.8	Emarginated	Present	Present	Complete	Degenerated	iii, 7	iii, 5	i, 9–10	i, 7	15–16	No	Yes	–
41	*T.zhenfengensis*	Entire body	Normal	7.1–16.7	22.2–34.5	Truncated	–	Present	Complete	Degenerated	iii, 7	iii, 5	i, 9	i, 5–7	14–15	No	No	4 + 36

In August and December 2023, we collected several specimens of *Triplophysa*, identified by the closely set anterior and posterior nostrils, while conducting a survey of cave fishes in western Guizhou Province, China. Morphological examination and molecular phylogenetic analysis indicated that these specimens were distinct from the 39 hypogean species of *Triplophysa*. We formally describe two new species, *Triplophysaziyunensis* sp. nov., and *Triplophysayaluwang* sp. nov., based on evidence from morphology, mitochondrial, and nuclear genes.

## ﻿Materials and methods

### ﻿Sampling

Thirty-seven samples of six species were collected in total for morphology comparison and genetic analysis (Fig. 1). Of these five specimens representing the new species, we collected *Triplophysaziyunensis* sp. nov., from Maoying Town, Ziyun County, Guizhou, and five specimens representing the new species, *Triplophysayaluwang* sp. nov., from Maoying Town, Ziyun County, Guizhou. The following specimens were used for morphometric data: nine specimens were *T.rosa* from Huolu Town, Wulong County, Chongqing; three specimens were *T.wudangensis* from Wudang District, Guiyang City, Guizhou; eight specimens were *T.qingzhenensis* from Qingzhen County, Guiyang City, Guizhou; and seven specimens were *T.guizhouensis* from Baijin Town, Huishui County, Guizhou. All of the specimens were fixed in 10% buffered formalin and later transferred to 75% ethanol for preservation. Muscle samples used for molecular analysis were preserved in 95% alcohol and stored at −20 °C. All of the specimens were deposited at Guizhou Normal University (GZNU), Guiyang City, Guizhou Province, China.

### ﻿DNA extraction, PCR, and sequencing

Genomic DNA was extracted from muscle tissue using a DNA extraction kit from Tiangen Biotech (Beijing) Co. Ltd. In total, six tissue samples used for molecular analysis were amplified and sequenced for mitochondrial gene cytochrome b (Cyt *b*) using the primers L3975 (5’-CGCCTGTTTACCAAAAACAT-3’) and H4551 (5’-CCGGTCTGAACTCAGATCACGT-3’) following Xiao et al. (2001). We also amplified one nuclear gene recombinase-activating 1 protein gene (*RAG1*) for 16 tissue samples, using primer LTF1 (5’-ATCATCGATGGCCTCTCAGGTT-3’) and LTR1 (5’-ACGTGGGCTAGAGTCTTGTGTAGGT-3’). PCR amplifications were performed within a 20 μl reaction volume with the cycling conditions that follow: An initial denaturing step at 95 °C for 4 min, 35 cycles of denaturing at 95 °C for 30 s, annealing at 45 °C (for Cyt b)/ 52 °C (for *RAG1*) for 40 s, and extension at 72 °C for 1 min followed by a final extension at 72 °C for 10 min. PCR products were purified with spin columns. The products were sequenced on an ABI Prism 3730 automated DNA sequencer at Chengdu TSING KE Biological Technology Co. Ltd. (Chengdu, China). All of the newly obtained sequences were submitted to GenBank (Table 3).

**Table 3.** Localities, voucher information, and GenBank numbers for all samples used. Numbers in bold were generated in this study.

ID	Species	Localities (* type localities)	Voucher ID	Cytb	RAG1
1	*T.guizhouensis*	Lewang Town, Wangmo County, Guzihou, China	GZNU20220313001	OQ241174	PQ117091
2	*T.guizhouensis*	Lewang Town, Wangmo County, Guzihou, China	gznu09	KU987438	PQ117092
3	*T.guizhouensis*	Baijin Town, Huishui County, Guzihou, China*	GZNU20230722007	GZ01	PQ117093
4	*T.yaluwang* sp. nov.	Maoying Town, Ziyun City, Guizhou, China*	GZNU20240118005	PQ117067	PQ117090
5	*T.yaluwang* sp. nov.	Maoying Town, Ziyun City, Guizhou, China*	GZNU20240118006	PQ117068	PQ117089
6	*T.longliensis*	/	SWU2016090300	MW582825	
7	*T.sanduensis*	Zhonghe Town, Sandu County, Guizhou, China*	SWU20170613001	MW582822	
8	*T.qini*	Houping Village, Wulong County, Chongqing, China*		ON528184	
9	*T.xiangxiensis*	Feihu Cave, Hunan, China*	/	JN696407	
10	*T.xiangxiensis*	/	IHB 2015010002	KT751089	
11	*T.nandanensis*	Hechi City, Guangxi,China	SWU20151123046	MG697588	
12	*T.nandanensis*	Liuzhai Town, Nandan County, Guangxi, China*	GZNU20230404005	OQ754126	
13	*T.nandanensis*	Liuzhai Town, Nandan County, Guangxi, China*	GZNU20230404007	OQ754128	
14	*T.tianeensis*	/	/	MW582826	
15	*T.tianeensis*	Bala Township, Tian ‘e County, Guangxi, China*	GZNU20230404003	OQ754124	
16	*T.nasobarbatula*	Dongtang Township, Libo County, Guizhou, China*	GZNU20190114001	MH685911	
17	*T.nasobarbatula*	Dongtang Township, Libo County, Guizhou, China*	GZNU20220313010	OQ241175	
18	*T.nasobarbatula*	Dongtang Township, Libo County, Guizhou, China*	GZNU20220313011	OQ241176	
19	*T.macrocephala*	Lihu Town, Nandan County, Guangxi, China*	GZNU20230404002	OQ754123	
20	*T.rosa*	Huolu Town, Wulong County, Chongqing, China*	SWU10100503	JF268621	
21	*T.rosa*	/	F3911	MG697587	
25	*T.rosa*	HuoLuTown, Wulong County, Chongqing City, China*	GZNU20230404009	OQ754130	PQ117076
22	*T.rosa*	Huolu Town, Wulong County, Chongqing, China*			PQ117079
23	*T.rosa*	Huolu Town, Wulong County, Chongqing, China*			PQ117080
24	*T.rosa*	Huolu Town, Wulong County, Chongqing, China*			PQ117081
26	*T.qingzhenensis*	Qingzhen County, Guiyang City, Guizhou, China*	IHB 201911150004	MT700458	
27	*T.qingzhenensis*	Qingzhen County, Guiyang City, Guizhou, China*			PQ117082
28	*T.qingzhenensis*	Qingzhen County, Guiyang City, Guizhou, China*			PQ117083
29	*T.qingzhenensis*	Qingzhen County, Guiyang City, Guizhou, China*			PQ117084
30	*T.wudangensis*	Wudang District, Guiyang City, Guizhou, China*	IHB 201908090003	MT700460	
31	*T.wudangensis*	Wudang District, Guiyang City, Guizhou, China*	GZNU20230404010	OQ754131	PQ117085
32	*T.wudangensis*	Wudang District, Guiyang City, Guizhou, China*			PQ117086
33	*T.wudangensis*	Wudang District, Guiyang City, Guizhou, China*			PQ117087
34	*T.wudangensis*	Wudang District, Guiyang City, Guizhou, China*			PQ117071
35	*T.ziyunensis* sp. nov.	Maoying Town, Ziyun City, Guizhou, China*	GZNU20230529003	PQ117069	PQ117072
36	*T.ziyunensis* sp. nov.	Maoying Town, Ziyun City, Guizhou, China*	GZNU20230529004	PQ117069	PQ117073
37	*T.ziyunensis* sp. nov.	Maoying Town, Ziyun City, Guizhou, China*	GZNU20230529005	PQ117071	PQ117074
38	*T.ziyunensis* sp. nov.	Maoying Town, Ziyun City, Guizhou, China*			PQ117075
39	*T.erythraea*	Dalong Cave, Huayuan County, Hunan, China*	/	MG967615	
40	*T.xuanweiensis*	Tangtang Town, Xuanwei City, Yunnan, China*	ASIZB223818	OL675196	
41	*T.xuanweiensis*	Tangtang Town, Xuanwei City, Yunnan, China*	ASIZB223819	OL675197	
42	*T.xuanweiensis*	Tangtang Town, Xuanwei City, Yunnan, China*	ASIZB223820	OL675198	
43	*T.zhenfengensis*	Xinlongchang Town, Xingren City, Guizhou, China*	GZNU20220313007	OQ241177	
44	*T.zhenfengensis*	Xinlongchang Town, Xingren City, Guizhou, China*	GZNU20220313008	OQ241178	
45	*T.zhenfengensis*	Xinlongchang Town, Xingren City, Guizhou, China*	GZNU20220313009	OQ241179	
46	*T.zhenfengensis*	Xinlongchang Town, Xingren City, Guizhou, China*	GZNU20220313005	OQ241180	
47	*T.rongduensis*	Rongbei Town, Ceheng County, Guzihou, China*	GZNU20230110001	OQ754135	
48	*T.rongduensis*	Rongbei Town, Ceheng County, Guzihou, China*	GZNU20230110002	OQ754136	
49	*T.rongduensis*	Rongbei Town, Ceheng County, Guzihou, China*	GZNU20230110003	OQ754137	
50	*T.anlongensis*	Xinglong Town, Anlong County, Guzihou, China*	GZNU20230112001	OQ754138	
51	*T.anlongensis*	Xinglong Town, Anlong County, Guzihou, China*	GZNU20230112002	OQ754139	
52	*T.anlongensis*	Xinglong Town, Anlong County, Guzihou, China*	GZNU20230112003	OQ754140	
53	*T.baotianensis*	Baotian Town, Panzhou City, Guzihou, China*	GZNU20180421005	MT992550	
54	*T.baotianensis*	Baotian Town, Panzhou City, Guzihou, China*	GZNU20180421006	OQ241181	
55	*T.panzhouensis*	Hongguo Town, Panzhou City, Guizhou, China*	GZNU20220513001	OQ754119	
56	*T.panzhouensis*	Hongguo Town, Panzhou City, Guizhou, China*	GZNU20220513002	OQ754120	
57	*T.panzhouensis*	Hongguo Town, Panzhou City, Guizhou, China*	GZNU20220513003	OQ754121	
58	*T.cehengensis*	Rongbei Town, Ceheng County, Guzihou, China*	GZNU20230109001	OQ754132	
59	*T.cehengensis*	Rongbei Town, Ceheng County, Guzihou, China*	GZNU20230109002	OQ754133	
60	*T.cehengensis*	Rongbei Town, Ceheng County, Guzihou, China*	GZNU20230109003	OQ754134	
61	*T.huapingensis*	/	F3917	MG697589	
62	*T.huapingensis*	Huaping Town, Leye County, Guangxi, China*	GZNU20230404004	OQ754125	
63	*T.langpingensis*	Longping Township, Tianlin County, Guangxi*	GZNU20230404001	OQ754122	
64	*T.qiubeiensis*	NijiaoVillage, Qiubei County, Yunnan , China*	GZNU20230404006	OQ754127	
65	*T.wulongensis*	Wulong County, Chongqing, China*	/	MW582823	
66	*T.wulongensis*	HuoLuTown, Wulong County, Chongqing City, China	GZNU20230404008	OQ754129	
67	*T.nujiangensa*	Fugong County, Yunnan, China	IHB201315814	KT213598	
68	*T.tibetana*	Mafamu lake, Xinjiang, China	NWIPB1106069	KT224364	
69	*T.tenuis*	Niutou river, Qingshui County, Gansu, China	IHB0917490	KT224363	
70	*T.wuweiensis*	Yongchang County, Gansu, China	IHB201307124	KT224365	
71	*Barbatulabarbatula*	/	/	KP715096	
72	*Barbatulalabiata*	Xinyuan County, Xinjiang, China	IHB201306569	KT192057	
73	*Homatulaberezowskii*	Qujing City, Yunnan, China	FS-2014-Y03	NC_040302	

### ﻿Phylogenetic analyses and nuclear haplotyping

Sixty-three mitochondrial Cyt *b* sequences, including six newly sequenced and 57 downloaded from GenBank, were used for molecular analysis. We followed the phylogenetic study of Luo et al. (2023) and used *Barbatulalabia*, *B.barbatula*, and *Homatulaberezowskii* as outgroups (Table 3).

All of the sequences were assembled and aligned using the MUSCLE (Edgar 2004) module in MEGA v. 7.0 (Kumar et al. 2016) with default settings. Alignment results were checked visually. Phylogenetic trees were constructed via both maximum likelihood (ML) and Bayesian inference (BI) methods. The ML was conducted in IQ-TREE v. 2.0.4 (Nguyen et al. 2015) with 10,000 ultrafast bootstrap (UBP) replicates (Hoang et al. 2018), and it was performed until a correlation coefficient of at least 0.99 was reached. The BI was performed in MrBayes v. 3.2.1 (Ronquist et al. 2012), and the best-fit model was obtained based on the Bayesian information criterion computed with PartitionFinder v. 2.1.1 (Lanfear et al. 2017). The first, second, and third codons of Cyt *b* were defined in this analysis.

The analysis suggested the best partition scheme for each codon position of Cyt *b.* TRNEF+I+G, HKY+I, and TIM+I+G were selected for the first, second, and third codons, respectively. Two independent runs were conducted in the BI analysis, each of which was performed for 2× 10^7^ generations and sampled every 1000 generations. The first 25% of the samples were discarded as a burn-in, resulting in a potential scale reduction factor of < 0.01. Nodes in the trees were considered well-supported when Bayesian posterior probabilities (BPP) were ≥ 0.95 and the ML ultrafast bootstrap value (UBP) was ≥ 95%. Uncorrected *p*-distances (1000 replicates) based on Cyt *b* were estimated using MEGA v. 7.0.

We also used the nuclear gene (*RAG1*) in PopART v. 1.7 (Leigh and Bryant 2015) based on the Median Joining method (Bandelt et al. 1999) to obtain haplotypes for assessing differences between the new species and genetically similar species.

### ﻿Morphometrics, comparisons, and statistics

Morphometric data were collected from 37 well-preserved specimens of *Triplophysa* (Suppl. material 1). Twenty measurements were recorded to the nearest 0.1 mm with digital calipers following the protocols of Tang et al. (2012) and Li et al. (2018). All of the measurements were taken on the left side when looking directly at the head end of the fish.

Comparative data for the 39 hypogean species of *Triplophysa* were obtained from the literature and specimen examination (Table 2). Specimens of 19 species from the type locality were collected and examined, and these included: *T.anlongensis*, *T.cehengensis*, *T.baotianensis*, *T.erythraea*, *T.guizhouensis*, *T.huapingensis*, *T.langpingensis*, *T.macrocephala*, *T.nasobarbatula*, *T.nandanensis*, *T.panzhouensis*, *T.qingzhenensis*, *T.qini*, *T.qiubeiensis*, *T.rosa*, *T.rongduensis*, *T.tianeensis*, *T.wudangensis*, and *T.zhenfengensis* (see Suppl. material 1). The measurements of these species were also included in the statistical analysis, taking into consideration the morphological similarity, genetic differences, and geographical distances of the two new species to *T.rosa*, *T.qingzhenensis*, *T.wudangensis*, *T.guizhouensis*, *T.sanduensis*, and *T.longliensis*.

Principal component analyses (PCAs) of size-corrected measurements and simple bivariate scatterplots were used to characterize the morphometric differences between the new species and closely related species. Mann–Whitney *U* tests were used to determine the significance of differences in morphometric characteristics between the new species and similar species. All of the statistical analyses were performed using SPSS 21.0 (SPSS, Inc., Chicago, IL, USA), and differences were considered statistically significant at *P* < 0.05. PCAs of morphological data were performed after logarithmic transformation and under nonrotational conditions. All of the pre-processing of morphological data was performed in Microsoft Excel (Microsoft Corporation 2016).

## ﻿Results

### ﻿Phylogenetic analyses, genetic divergence, and nuclear haplotypes

**Figure 2.**
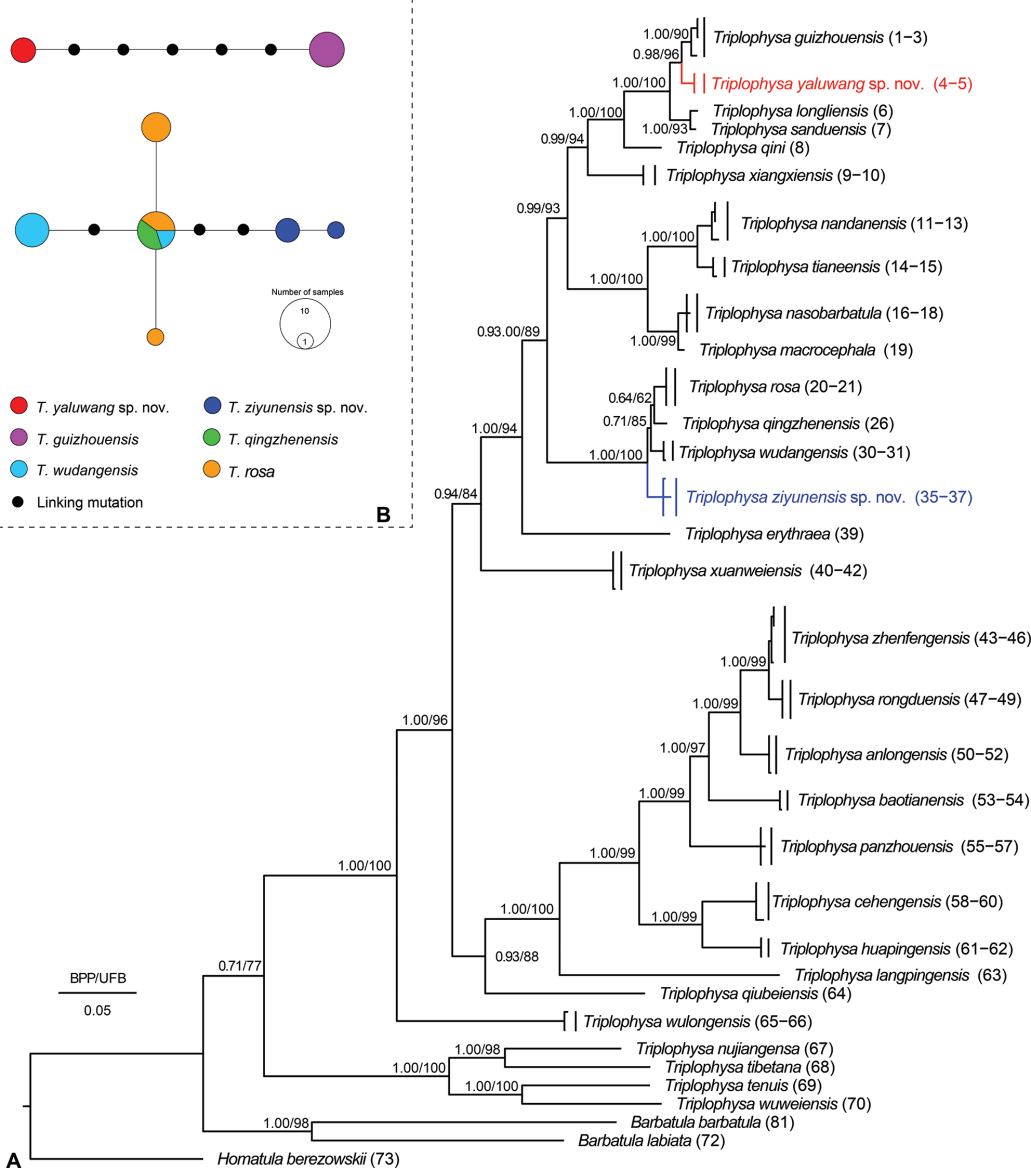
Phylogeny and nuclear gene haplotypes **A** phylogenetic tree based on mitochondrial Cyt *b* (1140 bp). Bayesian posterior probabilities (BPP) from BI analysis/ultrafast bootstrap supports (UBP) from ML analysis are noted beside nodes. Scale bars represent 0.05 nucleotide substitutions per site. The numbers at the tips of species name correspond to the ID numbers listed in Table 2**B** haplotypes inferred based on the nuclear gene *RAG1*.

ML and BI phylogenies were constructed based on mitochondrial Cyt *b*, with the sequence length being 1140 base pairs. The BI and ML phylogenetic trees showed a highly consistent topology that strongly supported the monophyly of the genus *Triplophysa*, and indicated that *Triplophysa* could be divided into two major clades, namely, the hypogean group and the epigean group (Fig. 2A).

The hypogean group contains 24 species from the karsts of southwest China (Chongqing, Guangxi, Guizhou, Hubei, and Yunnan) and two other lineages from western Guizhou that can be further divided into three clades (Fig. 2A): Clade A, only *T.wulongensis*, mainly in the Wujiang River basin (Fig. 1); subclade B1, including *T.qiubeiensis*, *T.langpingensis*, *T.huapingensis*, *T.cehengensis*, *T.panzhouensis*, *T.baotianensis*, *T.anlongensis*, *T.rongduensis*, and *T.zhenfengensis*, mainly in the Nampanjiang, Beipanjiang, and Hongshui River basins (Fig. 2A); and subclade B2 including *T.xuanweiensis*, *T.erythraea*, *T.wudangensis*, *T.qingzhenensis*, *T.rosa*, *T.macrocephala*, *T.nasobarbatula*, *T.tianeensis*, *T.nandanensis*, *T.xiangxiensis*, *T.qini*, *T.sanduensis*, *T.longliensis*, *T.guizhouensis*, and two other lineages from western Guizhou, mostly upstream of the Pearl and Yangtze rivers (Fig. 1).

All of the samples within subclade B1 from Shuitang Village, Maoying Town, Ziyun County, Guizhou Province (samples 35–38 in Table 3), clustered together in a sister clade to *T.wudangensis*, *T.qingzhenensis*, and *T.rosa* with strong node support (BPP/UBP = 1.00/1.00). This population could be distinguished from all of the known species and other undescribed lineages in this study via distinct morphological characteristics and molecular differences, with a lower *p*-distance of 1.8–2.0% (vs *T.wudangensis*, *T.rosa*, and *T.qingzhenensis*) (Table 4). Thus, the population at this locality represents an independently evolved lineage and is described below as a new species, *Triplophysaziyunensis* sp. nov.

**Table 4.** Uncorrected *p*-distance (%) between new species and 24 congeneric species of the genus *Triplophysa* based on mitochondrial Cyt *b*.

ID	Species	1	2	3	4	5	6	7	8	9	10	11	12	13	14	15	16	17	18	19	20	21	22	23	24	25
1	*T.ziyunensis* sp. nov.																									
2	*T.yaluwang* sp. nov.	9.5																								
3	*T.anlongensis*	15.3	14.5																							
4	*T.baotianensis*	14.9	14.0	6.8																						
5	*T.cehengensis*	15.1	13.8	3.8	6.8																					
6	*T.erythraea*	11.5	10.3	14.6	13.8	14.6																				
7	*T.guizhouensis*	9.8	1.4	14.7	14.5	13.9	10.6																			
8	*T.huapingensis*	15.1	14.1	9.9	10.2	9.3	14.9	14.6																		
9	*T.langpingensis*	14.2	14.7	14.1	14.0	13.8	15.8	15.1	14.4																	
10	*T.longliensis*	10.0	2.5	14.6	14.2	14.2	10.6	2.8	14.6	15.0																
11	*T.macrocephala*	10.0	9.2	15.4	15.9	15.3	12.0	9.6	14.9	15.7	9.4															
12	*T.microphthalmus*	14.5	13.8	10.0	11.0	9.5	15.1	13.8	5.9	13.8	14.0	15.1														
13	*T.nandanensis*	10.9	9.7	16.5	16.3	16.0	12.3	10.1	16.0	16.4	10.3	5.3	16.0													
14	*T.nasobarbatula*	9.9	9.1	15.5	15.8	15.2	12.1	9.6	14.9	15.3	9.4	0.9	15.2	5.5												
15	*T.panzhouensis*	15.8	13.6	7.0	7.7	8.2	13.4	14.3	10.1	14.7	12.9	15.3	10.2	15.7	15.2											
16	*T.qingzhenensis*	2.0	8.5	15.4	15.1	15.3	11.3	8.9	14.6	14.4	9.1	9.7	14.3	10.2	9.5	15.2										
17	*T.qini*	9.5	5.1	14.7	15.5	14.5	10.7	5.0	15.1	15.6	5.3	9.1	13.9	10.5	9.4	14.3	8.8									
18	*T.qiubeiensis*	14.1	12.7	14.7	14.2	14.2	13.8	12.8	14.6	14.5	13.3	14.6	14.5	14.5	14.2	14.4	13.8	13.4								
19	*T.rosa*	1.9	8.7	15.4	15.1	15.4	11.6	9.0	15.3	14.5	9.3	10.0	14.6	10.7	9.7	15.5	1.4	9.1	13.9							
20	*T.sanduensis*	9.9	2.5	14.8	14.7	14.5	11.0	2.6	14.7	15.1	0.7	9.3	13.9	10.5	9.2	13.5	9.0	5.3	13.6	9.3						
21	*T.tianeensis*	10.6	9.8	16.6	16.5	16.5	11.6	10.2	16.1	16.2	10.5	5.1	16.3	2.0	5.3	15.8	10.0	10.0	14.6	10.6	10.6					
22	*T.wudangensis*	1.8	8.7	15.3	14.9	15.4	11.3	9.1	14.8	14.4	9.3	10.1	14.4	10.7	9.8	15.5	1.6	9.3	14.2	1.5	9.3	10.5				
23	*T.wulongensis*	13.7	14.1	16.9	16.6	16.5	15.4	13.9	17.7	15.5	13.5	15.1	16.5	14.8	15.1	16.3	13.6	13.4	15.5	13.8	13.4	14.6	13.8			
24	*T.xiangxiensis*	9.3	7.9	14.3	14.7	14.2	11.2	8.1	15.2	14.6	7.2	8.5	14.5	9.7	8.4	13.9	8.6	5.9	13.8	8.7	7.8	9.0	9.0	14.4		
25	*T.xuanweiensis*	11.3	11.1	14.8	14.2	14.8	11.9	11.6	14.4	14.0	11.8	11.5	14.5	11.7	11.4	14.1	11.5	11.4	12.3	11.6	11.7	12.0	11.2	14.2	11.4	
26	*T.zhenfengensis*	15.6	13.9	3.4	6.8	0.9	14.5	14.0	9.0	13.6	14.4	15.6	9.3	16.2	15.3	7.7	15.4	14.8	14.1	15.6	14.6	16.7	15.5	16.3	14.3	14.4

All of the samples within subclade B1 from Xinzhai Village, Maoying Town, Ziyun County, Guizhou Province (samples 4 and 5 in Table 3), clustered together in a sister clade to *T.guizhouensis* with strong node support (BPP/UBP = 0.98/0.96). This population could be distinguished from all of the known species and other undescribed lineages in this study by distinct morphological characteristics and molecular differences, with a lower *p*-distance of 1.4% (vs *T.guizhouensis*) (Table 4). Thus, the population at this locality represents an independently evolved lineage and is described below as a new species, *Triplophysayaluwang* sp. nov.

Haplotype networks based on *RAG1* showed that unique, non-shared haplotypes were observed in the two new species and multiple linking mutations occurred with closely related species (Fig. 2B). We observed shared haplotypes from among *T.qingzhenensis*, *T.rosa*, and *T.wudangensis* (Fig. 2B). More haplotype diversity was found within *T.rosa*, a pattern that may be related to higher genetic diversity and wider distribution.

### ﻿Morphological analyses

Mann-Whitney U tests revealed differences in several morphological characteristics among the two new species (*T.ziyunensis* sp. nov. and *T.yaluwang* sp. nov.), and between the new species and the closely related species (Table 5). These significantly different measurements were concentrated on the head, barbel, fins, and tail (Table 5). There are significant morphological differences only in eye diameter and pectoral-fin ray length for *Triplophysayaluwang* sp. nov. and *T.guizhouensis*.

**Table 5.** Morphological comparison of *Triplophysaziyunensis* sp. nov. (*TZ*), *Triplophysayaluwang* sp. nov. (*TY*), *T.wudangensis* (*TW*), *T.rosa* (*TR*), *T.qingzhenensis* (*TQ*), and *T.guizhouensis* (*TG*). All units in mm. *P*-values are at the 95% significance level.

	*T.ziyunensis* sp. nov.		*T.yaluwang* sp. nov.		*T.wudangensis*		*T.rosa*		*T.qingzhenensis*		*T.guizhouensis*		TY vs TG	TZ vs TW	TZ vs TR	TZ vs TQ
	Range	Mean ± SD	Range	Mean ± SD	Range	Mean ± SD	Range	Mean ± SD	Range	Mean ± SD	Range	Mean ± SD				
Total length	78.6–120.0	103.8 ± 16.3	66.5–99.4	77.4 ± 15.3	73.4–85.9	79.5 ± 6.3	62.3–130.8	91.8 ± 23.8	84.6–123.3	109.6 ± 14.0	54.9–88.1	75.5 ± 12.2	0.808	0.101	0.386	0.38
Standard length	63.3–100.1	85.2 ± 14.4	54.1–83.9	64.3 ± 13.7	59.8–66.8	63.7 ± 3.6	49.8–104.3	74.0 ± 19.0	72.2–103	91.7 ± 11.4	45.8–72.8	62.5 ± 9.9	0.935	0.101	0.317	0.38
Head length	17.4–26.2	22.5 ± 3.6	13.4–19.7	15.3 ± 2.8	11.5–12.9	12.3 ± 0.7	14.7–28.6	21.0 ± 4.8	15.8–24.4	21.2 ± 3.2	6.2–16.7	13.3 ± 3.7	0.372	0.025	0.463	0.464
Head depth	8.6–11.9	10.5 ± 1.5	6.1–9.8	7.4 ± 1.7	6.6–7.4	7.0 ± 0.4	6.8–16.1	10.4 ± 3.0	8.6–12.9	11.3 ± 1.7	5.8–8.3	7.2 ± 0.9	0.935	0.025	0.739	0.188
Head width	10.5–16.1	12.9 ± 2.6	6.9–12.4	9.0 ± 2.4	8.2–9.2	8.8 ± 0.5	8.8–17.2	11.8 ± 2.6	10.2–15.8	13.5 ± 2.0	7.1–10.9	9.4 ± 1.3	0.57	0.025	0.386	0.884
Snout length	8.6–12	10.4 ± 1.6	0.0–9.3	6.0 ± 3.7	6.2–7	6.6 ± 0.4	7.3–10.4	8.7 ± 1.3	7.9–12.3	10.7 ± 1.7	5.2–7.4	6.4 ± 0.8	0.935	0.025	0.142	0.941
Eye diameter	0.4–1.2	0.9 ± 0.3	0.0–1.1	0.7 ± 0.4	0.6–0.7	0.6 ± 0.1	0.0–0.4	0.0 ± 0.1	0.2–0.3	0.2 ± 0.1	1.7–2.3	1.9 ± 0.2	0.004	0.227	0.001	0.003
Interorbital distance	4.5–6.5	5.4 ± 0.9	0.0–5	3.2 ± 1.9	4.0–4.5	4.3 ± 0.2	4.7–6.8	5.7 ± 0.9	5.1–7.7	6.8 ± 1.0	2.1–4.3	3.3 ± 0.9	0.685	0.025	0.327	0.028
Body depth	9.4–14.7	12.2 ± 2.3	6.5–13.5	9.1 ± 2.8	6.6–7.3	7.0 ± 0.4	5.9–17.2	10.2 ± 3.9	11.7–17	14.8 ± 1.9	6.3–11.2	8.8 ± 1.7	0.935	0.025	0.162	0.057
Body width	7.6–13.2	10.6 ± 2.5	8.1–9.1	8.5 ± 0.4	4.8–5.4	5.1 ± 0.3	4.3–14.7	7.9 ± 3.4	10.6–15.6	13.3 ± 1.9	5.8–9.8	7.3 ± 1.4	0.062	0.025	0.096	0.057
Maxillary barbel length	6.0–9.5	8.5 ± 1.5	3.0–6.8	4.9 ± 1.4	5.2–5.9	5.6 ± 0.3	5.1–10.3	7.5 ± 2.0	5.5–9.5	7.6 ± 1.3	3.9–6.4	5.2 ± 0.8	0.745	0.025	0.549	0.188
Outrostral barbel length	10.1–14.5	12.1 ± 1.8	5.3–8.1	6.4 ± 1.3	7.2–8.0	7.6 ± 0.4	6.5–10.6	8.4 ± 1.5	6.7–10.6	8.9 ± 1.3	4.6–7.7	6.2 ± 1.0	0.935	0.025	0.006	0.008
Inrostral barbel length	4.6–7	5.7 ± 0.9	2.2–4.5	3.5 ± 1.0	3.1–3.5	3.3 ± 0.2	3.2–6.5	4.5 ± 1.1	3.8–5.4	4.7 ± 0.6	2.8–3.9	3.3 ± 0.4	0.465	0.025	0.039	0.028
Dorsal-fin length	15.0–22.6	19.2 ± 3.2	11.1–16.1	13.0 ± 2.3	28.3–31.7	30.2 ± 1.7	13.4–26.9	18.2 ± 4.3	12.3–20.1	16.8 ± 2.9	9.0–14.4	12.6 ± 1.8	0.935	0.025	0.463	0.242
Dorsal-fin base length	9.6–13.4	11.8 ± 1.6	5.9–10.3	7.8 ± 1.8	7.3–8.2	7.8 ± 0.5	7.4–15.8	10.7 ± 3.2	7.9–9.6	8.8 ± 0.6	5.6–9.5	8.1 ± 1.3	0.808	0.025	0.463	0.005
Pectoral-fin length	13.7–21.7	18.5 ± 3.0	11.3–16.8	13.6 ± 2.2	12–13.4	12.8 ± 0.7	14.4–35.9	21.0 ± 6.6	14.3–21.7	17.9 ± 2.6	7.4–12.2	10.3 ± 1.6	0.019	0.025	0.386	0.661
Anal-fin length	11.9–18.2	15.6 ± 2.6	9.2–13.5	10.7 ± 1.9	9.8–10.9	10.4 ± 0.6	9.5–25.3	15.8 ± 4.8	10.2–16.1	14.0 ± 2.0	8.0–11.5	10.2 ± 1.2	0.935	0.025	0.841	0.188
Pelvic-fin length	11.6–19.3	15.3 ± 2.8	9.0–13.3	10.5 ± 1.9	9.2–10.2	9.8 ± 0.5	8.9–24.4	15.1 ± 4.8	10.8–16.8	14.0 ± 2.1	9.0–14.7	12.0 ± 1.8	0.372	0.025	0.641	0.38
Caudal peduncle length	9.8–16.9	13.6 ± 3.0	9.8–16.5	12.0 ± 2.8	11.8–13.1	12.5 ± 0.7	8.5–17.7	11.9 ± 3.5	11.9–18.2	15.9 ± 2.4	6.6–12.8	9.9 ± 2.4	0.291	0.655	0.257	0.107
Caudal peduncle depth	4.8–6.7	5.8 ± 0.8	3.8–6.6	4.8 ± 1.2	3.9–4.4	4.2 ± 0.2	3.1–9.3	5.5 ± 2.2	6.5–9.4	7.9 ± 1.0	3.5–5.9	4.8 ± 1.0	0.871	0.025	0.205	0.005

**Figure 3.**
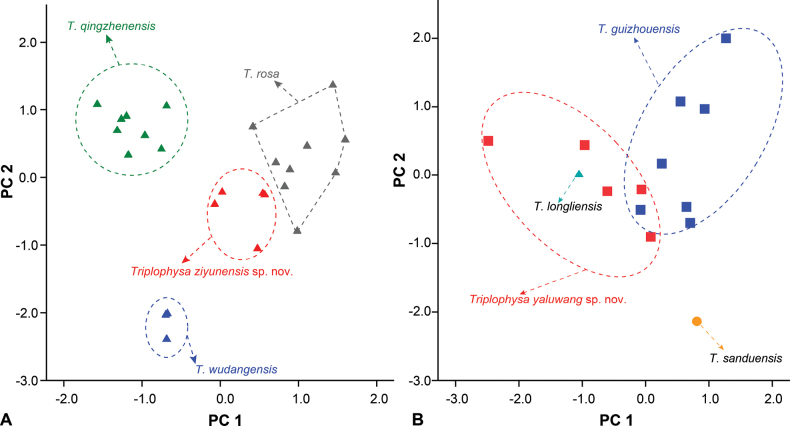
Plots of principal components analysis scores of **A***T.ziyunensis* sp. nov. and **B***T.yaluwang* sp. nov., and closely related species based on morphometric data.

Four principal component factors with eigenvalues greater than one were extracted based on the PCA of the morphological data. These factors accounted for 83.42% and 74.86% of the total variation (Suppl. material 2). The first principal component (PC1) accounted for 38.23% and 28.77% of the variation and was positively correlated with all of the variables (eigenvalue = 3.0 and 4.1). On the two-dimensional plots of PC1 and PC2, the new species *T.ziyunensis* sp. nov. can be readily distinguished from *T.wudangensis*, *T.rosa*, and *T.qingzhenensis* (Fig. 3A). *T.yaluwang* sp. nov. can be readily distinguished from *T.guizhouensis* (Fig. 3B), while the holotype of *T.longliensis* are mosaic in the *T.yaluwang* sp. nov. The two new species are clearly distinguished by morphological characteristics from the geographical and morphological relative species based on statistical analysis of the measurements and the PCA result.

### ﻿Taxonomic account

#### ﻿ Triplophysa ziyunensis

Taxon classificationAnimaliaCypriniformesNemacheilidae

Wu, Luo, Xiao & Zhou sp. nov.

7DAAB8B9-EF6F-58E9-98D1-56EC831C3061

https://zoobank.org/BA4F7B39-A976-4D59-AE66-439DB9130A2E

**Figure 4.**
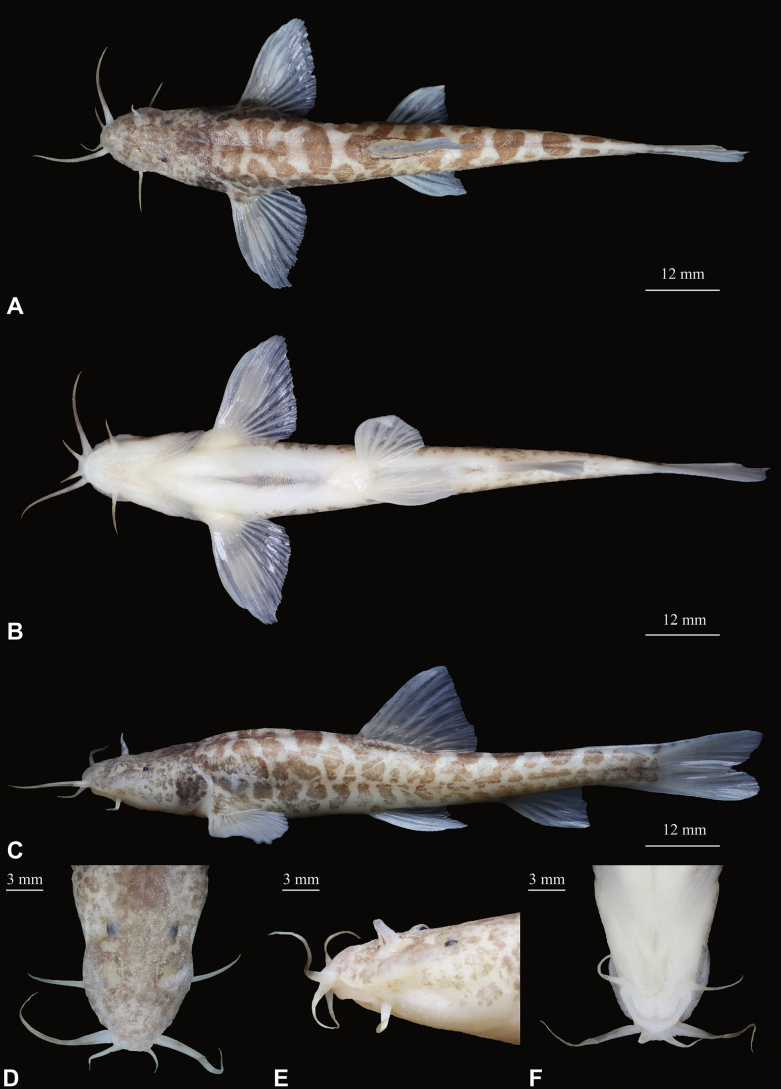
Morphological characteristics of holotype GZNU20230529001 of *Triplophysaziyunensis* sp. nov. in preservative (10% formalin) **A** dorsal view **B** ventral view **C** lateral view **D** dorsal view of head **E** lateral view of head, and **F** ventral view of head.

**Figure 5.**
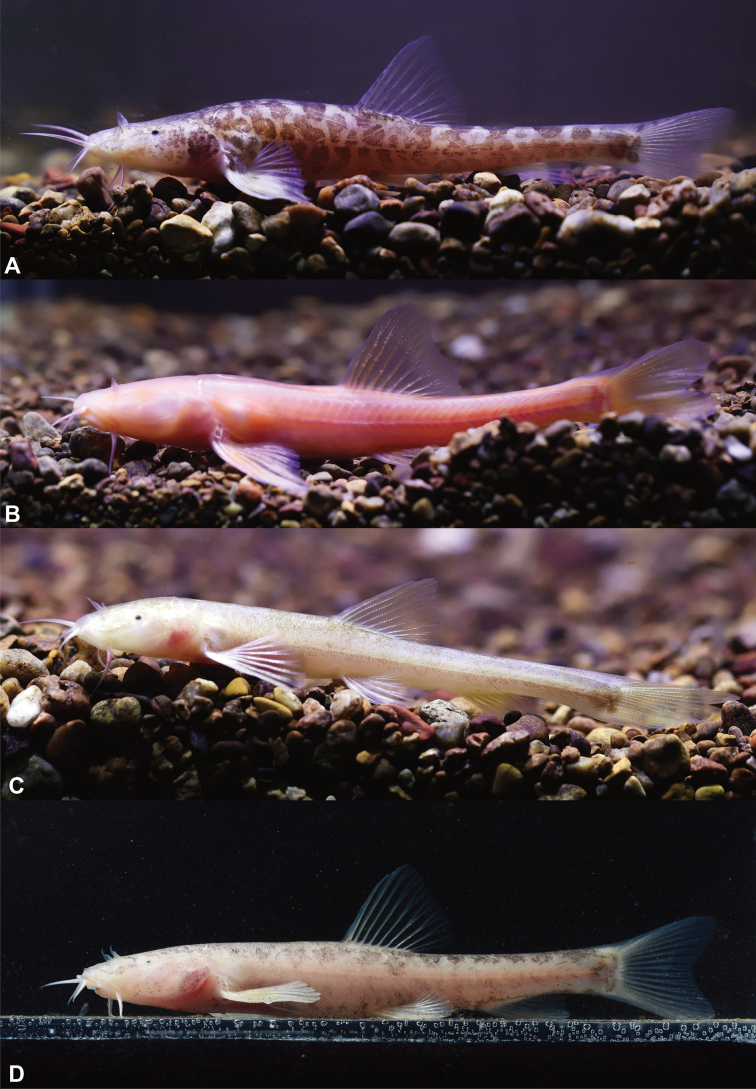
Ecological photographs of *Triplophysaziyunensis* sp. nov. and closely related species in life **A***Triplophysaziyunensis* sp. nov. **B***T.rosa***C***T.wudangensis*, and **D***T.qingzhenensis*, from Dr. Zhi-Xuan Zeng.

Figs 4 , 5 , Table 5 , Suppl. material 1

##### Type material.

***Holotype*.** GZNU20230529001 (Fig. 4), 105.1 mm total length (TL), 86.7 mm standard length (SL), collected by Li Wu and Xing-Liang Wang on 29 May 2023, at Shuitang Village, Maoying Town, Ziyun County, Guizhou Province, China (25.96846238°N, 106.13737106°E; 1228 m a.s.l.; Fig. 1).

***Paratypes*.** Four specimens from the same locality as the holotype: GZNU20230226008-226010, and GZNU20230529002, 63.3–100.1 mm SL, collected by Tao Luo, Li Wu, Xing-Liang Wang, Xin-Rui Zhao, and Chang-Ting Lan on 26 February 2023.

##### Diagnosis.

*Triplophysaziyunensis* sp. nov. is distinguished from other hypogean species of the genus *Triplophysa* by the following characteristics in combination: (1) body naked, scaleless, pigmented markings on surface of body, except ventral; (2) eyes reduced, diameter 2.4–4.9% of head length (HL); (3) pelvic-fin tip extending to anus; (4) tip of pectoral fin not reaching pelvic fin origin; (5) anterior and posterior nostrils closely set, with anterior nostril elongated to a barbel-like tip; (6) tip of outrostral barbel extending backward, not reaching anterior margin of eye; (7) lateral line complete; (8) posterior chamber of air bladder degenerated; and (9) dorsal-fin rays iii-8, pectoral-fin rays i-10, pelvic-fin rays i-6, anal-fin rays iii-5, and 16 branched caudal-fin rays.

##### Description.

Morphological data on the specimens of *Triplophysaziyunensis* sp. nov. are provided in Table 5 and Suppl. material 1. Body elongated and cylindrical, posterior portion gradually compressed from dorsal fin to caudal-fin base, with deepest body depth anterior to dorsal-fin origin, deepest body depth 13–16% of standard length (SL). Dorsal profile slightly convex from snout to dorsal-fin insertion, and then straight from posterior portion of dorsal-fin origin to caudal-fin base. Ventral profile flat. Head short, length 26–27% of SL, slightly depressed and flattened, width slightly greater than depth (head width (HW)/head depth (HD) = 1.1–1.3). Snout slightly pointed, and snout length 46–50% of HL. Mouth inferior and curved, mouth corner situated below anterior nostril, upper and lower lips smooth, lower lip with V-shaped median notch. Three pairs of barbels are present: inner rostral barbel long, length 23–27% of HL, backward extending to corner of the mouth; out rostral barbel long, length 52–58% of HL, backward extending to beyond posterior margin of eyes. Maxillary barbel not extending to posterior margin of operculum, length 34–42% of HL. Anterior and posterior nostrils closely set, length 0.20–0.25 mm. Anterior nostril tube long, with an elongated short barbel-like tip, tip of posterior nostril extending backward not reaching to anterior margin of the eye. Eyes reduced, with diameter 2–5% of HL. Gill opening small, gill rakers not developed, ten inner gill rakers on first gill arch (*n* = 1).

Dorsal-fin rays iii-8, pectoral-fin rays i-10, pelvic-fin rays i-6, anal-fin rays i-5, 16 branched caudal-fin rays. Dorsal fin short, length 20–23% of SL, distal margin emarginated, origin anterior to pelvic-fin insertion and situated slightly posterior to the midpoint between snout tip and caudal-fin base, first branched ray longest, shorter than head length, tip of dorsal fin vertical to the anus. Pectoral fin moderately developed, length 22–24% of SL, tip of pectoral fin extending backward almost to midpoint between origin of pectoral and pelvic fin origins, not reaching to pelvic fin origin. Pelvic fin length 16–20% of SL, vertically aligned with third branched ray of dorsal fin, tips of pelvic fin reaching anus. Anal fin length 16–20% of SL, distal margin truncated, origin close to anus, tips of anal fin not reaching caudal-fin base, distance between tips of anal fin and anus 8.5× the eye diameter. Caudal fin forked, upper lobe equal in length to lower lobe, tips pointed, caudal peduncle length ~ 13.6 mm, caudal peduncle depth ~ 5.8 mm, with weak adipose crests along both dorsal and ventral sides. Total vertebrae: 39 (*n* = 1).

Cephalic lateral line system developed. Lateral line complete, exceeding tip of pectoral fin and reaching base of caudal fin. Two chambers of air bladder, anterior chamber dumbbell-shaped and membranous, open on both sides, slightly closed posteriorly; posterior chamber degenerated, slightly filling the body cavity, connected with anterior chamber by a long, slender tube.

##### Coloration.

In cave water, the body of living fish is semi-translucent and pale pink, with irregular dark brownish brown patches on the head and body (Fig. 5). After fixation in 10% formalin solution, the body color was pale grey, and the dark-brown patches on the head and body were more prominent (Fig. 4).

##### Secondary sex characteristics.

No secondary sex characteristics were observed based on the present specimens of *Triplophysaziyunensis* sp. nov.

##### Comparisons.

Detailed comparative morphological data of *Triplophysaziyunensis* sp. nov. with the 39 recognized hypogean species of *Triplophysa* are given in Table 2. *Triplophysaziyunensis* sp. nov. is genetically close to *T.qingzhenensis*, *T.rosa*, and *T.wudangensis* and shares some similar morphological characters, such as reduced eye degeneration and degenerated body pigmentation, pigmented markings on the body surface, except ventral, but can still be distinguished by a combination of some morphological characters.

*Triplophysaziyunensis* sp. nov. is be distinguished from *T.qingzhenensis* and *T.wudangensis* by having 10 branched pectoral fin rays (vs 8–9), 6 branched pelvic-fin rays (vs 5), 16 branched caudal fin rays (vs 14–15), and inhabiting the Pearl River basin (vs Yangtze River basin).

*Triplophysaziyunensis* sp. nov. can be distinguished from *T.rosa* by having reduced body pigmentation, pigmented markings on body surface, except ventral (vs absence), eyes reduced, diameter 2.4–4.9% of HL (vs absent), 8 branched dorsal fin rays (vs 9), 10 branched pectoral fin rays (vs 12), 6 branched pelvic-fin rays (vs 7), 16 branched caudal fin rays (vs 14), and inhabiting the Pearl River basin (vs Yangtze River basin).

##### Ecology and distribution.

*Triplophysaziyunensis* sp. nov. has only been found in one cave in Shuitang Village, Maoying Town, Ziyun County, Guizhou Province, China, at an elevation of 1134 m. The pool where the new species was found is more than 15 m long, 13 m wide, and ~ 3 m deep, with a slow flow of water, and is located 80 m further inside the entrance of the cave. Inside the cave, another fish (*Sinocyclocheilusmultipunctatus*, three individuals), bats (*Rhinolophus* sp., five individuals), and frogs (*Odorranawuchuanensis*, 11 individuals) were found. Outside the cave, rapeseed and peppers were being grown. The population of the new species is very small and only five specimens were collected.

##### Remarks.

The new species, *Triplophysaziyunensis* sp. nov., inhabits the underground rivers of the type locality. Eyes are present and reduced, and with irregular dark brownish brown patches on the head and body. Therefore, this species can be considered as a stygophile fish within the hypogean group of the genus *Triplophysa*.

##### Etymology.

The specific epithet *ziyunensis* refers to the type locality of the new species: Shuitang Village, Maoying Town, Ziyun County. We propose the common English name “Ziyun high-plateau loach” and the Chinese name “Zǐ Yún Gāo Yuán Qīu (紫云高原鳅)”.

#### ﻿ Triplophysa yaluwang

Taxon classificationAnimaliaCypriniformesNemacheilidae

Lan, Liu, Zhou & Zhou sp. nov.

E615223B-F2B0-5FB6-96BD-9D895AE03E23

https://zoobank.org/DE306B1B-F770-4E79-9B9E-400CEC202266

**Figure 6.**
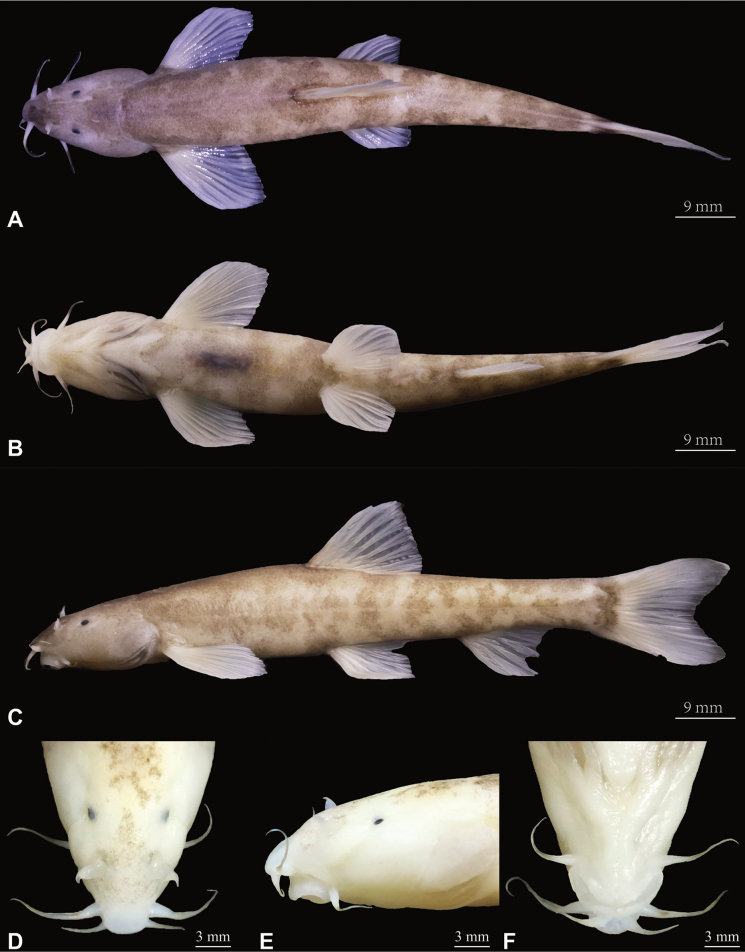
Morphological characteristics of holotype GZNU20240118001 of *Triplophysayaluwang* sp. nov. in preservative (10% formalin) **A** dorsal view **B** ventral view **C** lateral view **D** dorsal view of head **E** lateral view of head, and **F** ventral view of head.

**Figure 7.**
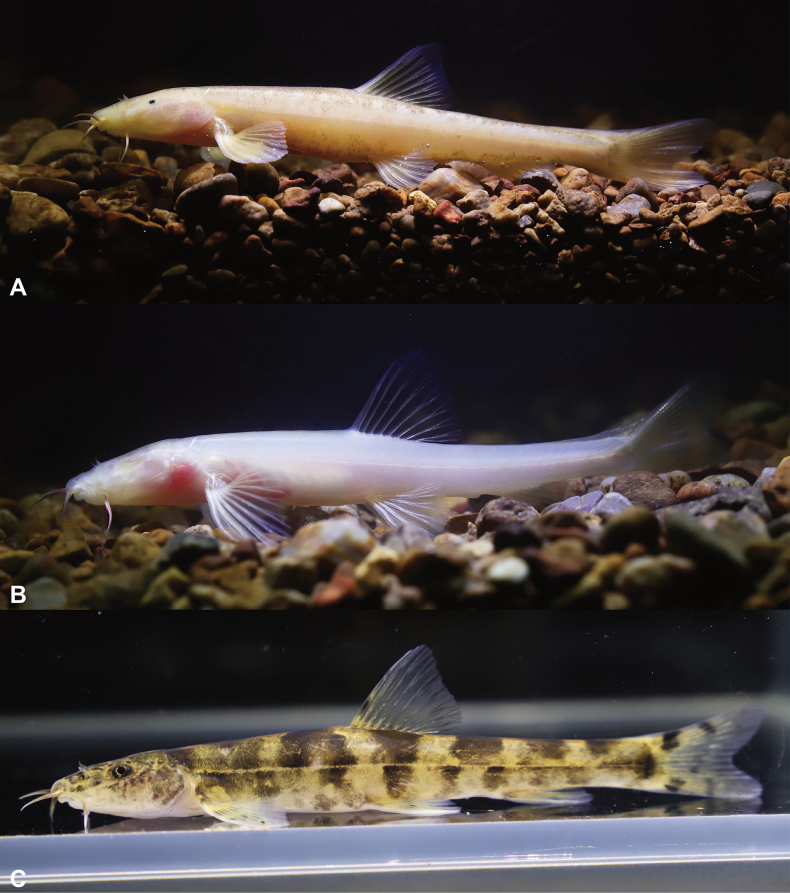
Ecological photographs of *Triplophysayaluwang* sp. nov. and closely related species in life **A***Triplophysayaluwang* sp. nov. (paratype, GZNU20240118002) **B***Triplophysayaluwang* sp. nov. (paratype, GZNU20240118005), and **C***T.guizhouensis*.

Figs 6 , 7 , Table 5 , Suppl. material 1

##### Type material.

***Holotype*.** GZNU20240118001 (Fig. 6), 87.6 mm total length (TL), 73.9 mm standard length (SL), collected by Jia-Jun Zhou on 18 January 2024, in Xinzhai Village, Maoying Town, Ziyun County, Guizhou Province, China (25.89908752°N, 106.07921141°E, 1276 m a.s.l.; Fig. 1).

***Paratypes*.** Four specimens from the same locality as the holotype: GZNU20240118002–118005, 54.1–83.9 mm SL, collected by Jia-Jun Zhou and Ye-Wei Liu on 27 September 2023.

##### Diagnosis.

*Triplophysayaluwang* sp. nov. is distinguished from other hypogean species of the genus *Triplophysa* by the following characteristics in combination: (1) body naked, scaleless, with irregular pale dark brownish brown markings, except ventral; (2) eyes reduced, diameter 4.6–6.1% of head length; (3) pelvic-fin tip reaching anus; (4) tip of pectoral fin not reaching to pelvic fin origin; (5) anterior and posterior nostrils closely set, with the anterior nostril elongated to a barbel-like tip; (6) tip of outrostral barbel extending backward, not reaching to anterior margin of eye; (7) lateral line complete; (8) posterior chamber of air bladder degenerated; and (9) dorsal-fin rays iii-7, pectoral-fin rays i-9, pelvic-fin rays i-5, anal-fin rays i-5, and 14 branched caudal-fin rays.

##### Description.

Morphological data of *Triplophysayaluwang* sp. nov. specimens are provided in Table 5 and Suppl. material 1. Body elongated and cylindrical, posterior portion gradually compressed from dorsal fin to caudal-fin base, with deepest body depth anterior to dorsal-fin origin, deepest body depth 12–16% of SL. Dorsal profile slightly convex from snout to dorsal-fin insertion, then straight from posterior portion of dorsal-fin origin to caudal-fin base. Ventral profile flat. Head short, length 26–27% of SL, slightly depressed and flattened, width slightly greater than depth (HW/HD = 1.1–1.3). Snout slightly pointed, and snout length 43–52% of HL. Mouth inferior and curved, mouth corner situated below anterior nostril, upper and lower lips smooth, lower lip with V-shaped median notch. Three pairs of barbels are present: inner rostral barbel long, length 16–27% of HL, backward extending to corner of mouth; out rostral barbel long, length 39–44% of HL, backward extending to beyond anterior margin of eyes. Maxillary barbel not extending to posterior margin of operculum, length 22–36% of HL. Anterior and posterior nostrils closely set, length 0.44–0.82 mm. Anterior nostril tube long, with an elongated short barbel-like tip, tip of posterior nostril extending backwards not reaching to anterior margin of eye. Eyes reduced, with diameter 5–6% of HL. Gill opening small, gill rakers not developed, nine inner gill rakers on first gill arch (*n* = 1).

Dorsal-fin rays iii-7, pectoral-fin rays i-9, pelvic-fin rays i-5–6, anal-fin rays i-5, 14 branched caudal-fin rays. Dorsal fin short, length 19–22% of SL, distal margin emarginated, origin anterior to pelvic-fin insertion and situated slightly posterior to the midpoint between snout tip and caudal-fin base, first branched ray longest, shorter than head length, tip of dorsal fin vertical to anus. Pectoral fin moderately developed, length 19–25% of SL, tip of pectoral fin extending backward almost to the midpoint between origin of pectoral and pelvic fin origins, not reaching to pelvic fin origin. Pelvic fin length 16–17% of SL, vertically aligned with second branched ray of dorsal fin, tips of pelvic fin reaching to anus. Anal fin length 16–18% of SL, distal margin truncated, origin close to anus, tips of anal fin not reaching caudal-fin base, distance between tips of anal fin and anus 2.2× the eye diameter. Caudal fin forked, upper lobe slightly longer than lower lobe, tips pointed, caudal peduncle length ~ 12 mm, caudal peduncle depth ~ 4.8 mm, with weak adipose crests along both dorsal and ventral sides. Total vertebrae: 40 (*n* = 1).

Cephalic lateral line system developed. Lateral line complete, exceeding tip of pectoral fin and reaching base of caudal fin. Two chambers of air bladder, anterior chamber dumbbell-shaped and membranous, open on both sides, slightly closed posteriorly; posterior chamber degenerated, slightly filling the body cavity, connected with anterior chamber by a long, slender tube.

##### Coloration.

In cave water, living fish were semi-translucent with a pale pink body with irregular dark brownish brown patches on the Entire body (Fig. 7). After fixation in 10% formalin, the body color was white, and the dark brown color lightened (Fig. 6).

##### Variations.

Among the five specimens collected, GZNU20240118002–118004 are essentially identical to the holotype in fin characteristics and body coloration. GZNU20240118005 differs from the holotype by the absence of body pigmentation and the absence of the eye (Fig. 7).

##### Secondary sex characteristics.

Secondary sex characteristics were not observed in the specimens of *Triplophysayaluwang* sp. nov.

##### Comparisons.

Detailed morphological comparative data of *Triplophysayaluwang* sp. nov. with *Triplophysaziyunensis* sp. nov. and the 39 hypogean species of *Triplophysa* are given in Table 2. *Triplophysayaluwang* sp. nov. is genetically close to *T.guizhouensis*, *T.longliensis*, and *T.sanduensis*, but it can be distinguished in combination with morphological characteristics.

*Triplophysayaluwang* sp. nov. can be distinguished from *Triplophysaziyunensis* sp. nov. by having dorsal fin distal margin being emarginated (vs truncated), total vertebrae 40 (vs 39), seven branched dorsal fin rays (vs 8), nine branched pectoral fin rays (vs 10), and 14 branched caudal fin rays (vs 16).

*Triplophysayaluwang* sp. nov. is distinguished from *T.longliensis* by having eyes reduced, small diameter 4.6–6.1% of HL (vs normal, diameter 9.5–11.5% of HL), interorbital width, 24.3–26.0% of HL (vs 31.4–37.5 of HL), total vertebrae 40 (vs 42), degenerated posterior chamber of air bladder (vs developed), seven branched dorsal-fin rays (vs 8), nine branched pectoral-fin rays (vs 10), and 14 branched caudal-fin rays (vs 15–16).

*Triplophysayaluwang* sp. nov. is distinguished from *T.sanduensis* by having eyes reduced, small diameter 4.6–6.1% of HL (vs normal, diameter 11.9–15.4% of HL), interorbital width, 24.3–26.0% of HL (vs 31.2–40.2 of HL), total vertebrae 40 (vs 41), dorsal-fin rays, iii, 7 (vs ii, 8–9), three unbranched anal-fin rays (vs 1), 14 branched caudal-fin rays (vs 17–18), and tip of pelvic fin reaching anus (vs not reaching anus).

*Triplophysayaluwang* sp. nov. differs from *T.guizhouensis* by having eyes reduced, diameter 4.6–6.1% of HL (vs normal, diameter 9.4–12.1% of HL), dorsal fin distal margin being emarginated (vs truncated), body scaleless (vs body covered by sparse scales), degenerated posterior chamber of air bladder (vs developed), seven branched dorsal fin rays (vs 8), five branched anal-fin rays (vs 6), and tip of pelvic fin reaching anus (vs not reaching anus).

##### Ecology and distribution.

The new species *Triplophysayaluwang* sp. nov. was found in one cave far from the village of Xinzhai Village, Maoying Town, Ziyun County, Guizhou Province, China (Fig. 1), in a water system where the underground river is a tributary of the Hongshui River. The cave habitat is a vertical shaft with an entrance located halfway up the mountainside. The underground river is approximately 150 m deep from the entrance, and the accessible portion is around 200 m long, 3 m wide, and 1–2 m deep. In this cave, the new species is sympatric with *Sinocyclocheilusmultipunctatus* and some unnamed spiders.

##### Remarks.

The new species, *Triplophysayaluwang* sp. nov., inhabits the underground rivers of the type locality. Eyes are present and reduced, and with irregular dark brownish brown patches on the head and body. Therefore, this species can be considered as a stygophile fish within the hypogean group of the genus *Triplophysa*.

##### Etymology.

The specific epithet *yaluwang* comes from King Yalu, a hero to the Miao people of Ziyun County, Guizhou Province, China, where the type locality is found. He was the 18^th^ generation leader of the Miao ancestors in western China and led the Miao people through many trials and tribulations. He eventually carved out a suitable land for his people to live in near the type locality. His deeds have been preserved in the form of a song, which has been organized into the first full-length heroic epic of the Hmong, King Yalu. We propose the common English name “King Yalu high-plateau loach” and the Chinese name “Yà Lǔ Wáng Gāo Yuán Qīu (亚鲁王高原鳅)”.

## ﻿Discussion

We describe two new species, *Triplophysaziyunensis* sp. nov. and *Triplophysayaluwang* sp. nov., based on morphological comparisons (Table 2), mitochondrial DNA sequence differences, and nuclear gene haplotypes (Fig. 2). The description of these two new species increases the number of species in the hypogean group of *Triplophysa* from 39 to 41 and the previous number of known species from Guizhou is increased to 15. The previous 13 species are *T.cehengensis*, *T.rongduensis*, *T.panzhouensis*, *T.anlongensis*, *T.baotianensis*, *T.guizhouensis*, *T.longliensis*, *T.nasobarbatula*, *T.qingzhenensis*, *T.sanduensis*, *T.wudangensis*, *T.wulongensis*, and *T.zhenfengensis* (Table 1). Our previous studies revealed that the cave-dwelling species of *Triplophysa* in Guizhou are concentrated in the south, central, and southwest (Fig. 1). Before this study, a large recording gap existed between central-southern and western Guizhou (Fig. 1), suggesting the possible presence of cryptic species in this region (Luo et al. 2023). This hypothesis was supported by the results of the present study. Similarly, discontinuities with previous records remain in northeastern and eastern Guizhou, suggesting that it would be useful to focus efforts there on the discovery of new species or new distribution areas.

The new species described here have only slight mitochondrial differences from closely related species (Table 4). For example, the new species *T.yaluwang* sp. nov. clusters with *T.guizhouensis*, both from the Hongshui River drainage basin, at a genetic distance of 1.4%. The type locality of the two new species is near the watershed between the Pearl and Yangtze rivers, i.e., the Miaoling Mountains (Fig. 1). Geological evidence suggests that the Miaoling mountains formed in the Early Pleistocene and eventually became the watershed between the Pearl River and Yangtze River systems during the mid- to late Pleistocene (Zhou and Chen 1993). Thus, the slight mitochondrial differences are associated with multiple connectivity between rivers, includes both surface and underground rivers, which leads to potential mitochondrial introgression (Yuan et al. 2023). Similar gene flow was observed among species of *Triplophysa* in the Qinghai-Tibetan Plateau region (Feng et al. 2019). This hypothesis is also supported by the mitochondrial matrilineal tree in this study, i.e., the species of the independent hydrological origin are not clustered together in the phylogenetic tree, but rather are distributed in a mosaic fashion (Figs 1, 2). However, this could also be related to short-term radial species formation. To evaluate this situation, we suggest the use of additional nuclear genetic markers or genomic evidence in the description of new species.

## Supplementary Material

XML Treatment for Triplophysa ziyunensis

XML Treatment for Triplophysa yaluwang

## ﻿Additional information

### Author ORCIDs

Chang-Ting Lan https://orcid.org/0009-0007-2381-3601

Li Wu https://orcid.org/0000-0002-7898-7517

Tao Luo https://orcid.org/0000-0003-4186-1192

Ye-Wei Liu https://orcid.org/0000-0003-4712-1072

Jia-Jun Zhou https://orcid.org/0000-0003-1038-1540

Jing Yu https://orcid.org/0009-0004-3629-3826

Xin-Rui Zhao https://orcid.org/0000-0002-9125-6276

Ning Xiao https://orcid.org/0000-0002-7240-6726

Jiang Zhou https://orcid.org/0000-0003-1560-8759

### Data availability

All of the data that support the findings of this study are available in the main text or Supplementary Information.

## Supplementary materials

Supplementary material 1 Morphological characters and measurement data of the new species *Triplophysaziyunensis* sp. nov., *Triplophysayaluwang* sp. nov., *T.wudangensis*, *T.rosa*, *T.qingzhenensis*, and *T.guizhouensis* Data type docx Explanation note *indicates the holotype. https://binary.pensoft.net/file/1150828This dataset is made available under the Open Database License (http://opendatacommons.org/licenses/odbl/1.0/). The Open Database License (ODbL) is a license agreement intended to allow users to freely share, modify, and use this Dataset while maintaining this same freedom for others, provided that the original source and author(s) are credited.Chang-Ting Lan, Li Wu, Tao Luo, Ye-Wei Liu, Jia-Jun Zhou, Jing Yu, Xin-Rui Zhao, Ning Xiao, Jiang Zhou

Supplementary material 2 Results and percentage of variance explained by principal component analysis Data type docx https://binary.pensoft.net/file/1150829This dataset is made available under the Open Database License (http://opendatacommons.org/licenses/odbl/1.0/). The Open Database License (ODbL) is a license agreement intended to allow users to freely share, modify, and use this Dataset while maintaining this same freedom for others, provided that the original source and author(s) are credited.Chang-Ting Lan, Li Wu, Tao Luo, Ye-Wei Liu, Jia-Jun Zhou, Jing Yu, Xin-Rui Zhao, Ning Xiao, Jiang Zhou

Supplementary material 3 Specimens examined in this work Data type docx https://binary.pensoft.net/file/1150830This dataset is made available under the Open Database License (http://opendatacommons.org/licenses/odbl/1.0/). The Open Database License (ODbL) is a license agreement intended to allow users to freely share, modify, and use this Dataset while maintaining this same freedom for others, provided that the original source and author(s) are credited.Chang-Ting Lan, Li Wu, Tao Luo, Ye-Wei Liu, Jia-Jun Zhou, Jing Yu, Xin-Rui Zhao, Ning Xiao, Jiang Zhou

## References

[B1] Bandelt HJ Forster P Röhl A (1999) Median-joining networks for inferring intraspecific phylogenies. Molecular Biology and Evolution 16(1): 37−48. 10.1093/oxfordjournals.molbev.a026036 10331250

[B2] Chen SJ Peng ZG (2019) *Triplophysasanduensis*, a new loach species of nemacheilid (Teleostei: Cypriniformes) from South China. Zootaxa 4560(2): 375–384. 10.11646/zootaxa.4560.2.10 31716587

[B3] Chen XY Yang JX (2005) *Triplophysarosa* sp. nov.: A new blind loach from China. Journal of Fish Biology 66(3): 599–608. 10.1111/j.0022-1112.2005.00622.x

[B4] Chen YR Yang JX Xu GC (1992) A new blind loach of *Triplophysa* from Yunnan stone forest with comments on its phylogenetic relationship. Zoological Research 13(1): 17–23. [In Chinese]

[B5] Chen XY Cui GH Yang JX (2004) A new cave dwelling fish species of genus *Triplophysa* (Balitoridae) from Guangxi, China. Zoological Research 25(3): 227–231. [In Chinese]

[B6] Chen S Sheraliev B Shu L Peng Z (2021) *Triplophysawulongensis*, a new species of cave-dwelling loach (Teleostei, Nemacheilidae) from Chongqing, Southwest China. ZooKeys 1026: 179–192. 10.3897/zookeys.1026.61570 33850421 PMC8018939

[B7] Chu XL Chen YR (1979) A new blind cobitid fish (Pisces, Cypriniformes) from subterranean waters in Yunnan, China. Dong Wu Xue Bao 25(3): 285–287. [In Chinese]

[B8] Chu XL Chen YR (1990) The fishes of Yunnan, China. Part 2. Science Press, Beijing, 313 pp. [In Chinese]

[B9] Deng SQ Wang XB Zhang E (2022) *Triplophysaqini*, a new stygobitic species of loach (Teleostei: Nemacheilidae) from the upper Chang-Jiang Basin in Chongqing, Southwest China. Ichthyological Exploration of Freshwaters 1178: 1–11. 10.23788/IEF-1178

[B10] Edgar RC (2004) MUSCLE: Multiple sequence alignment with high accuracy and high throughput. Nucleic Acids Research 32(5): 1792–1797. 10.1093/nar/gkh340 15034147 PMC390337

[B11] Feng C Zhou W Tang Y Gao Y Chen J Tong C Liu S Wanghe K Zhao K (2019) Molecular systematics of the *Triplophysarobusta* (Cobitoidea) complex: extensive gene flow in a depauperate lineage. Molecular Phylogenetics and Evolution 132: 275–283. 10.1016/j.ympev.2018.12.009 30550962

[B12] Fricke R Eschmeyer WN Van der Laan R (Eds) (2024) Catalog of fishes: genera, species, references. California Academy of Sciences, San Francisco. https://researcharchive.calacademy.org/research/ichthyology/catalog/fishcatmain.asp [accessed 17 January 2023]

[B13] He CL Zhang E Song ZB (2012) *Triplophysapseudostenura*, a new nemacheiline loach (Cypriniformes: Balitoridae) from the Yalong River of China. Zootaxa 3586(1): 272–280. 10.11646/zootaxa.3586.1.26

[B14] Hoang DT Chernomor O von Haeseler A Minh BQ Vinh L (2018) UFBoot2: Improving the ultrafast bootstrap approximation. Molecular Biology and Evolution 35(2): 518–522. 10.1093/molbev/msx281 29077904 PMC5850222

[B15] Huang TF Zhang PL Huang XL Wu T Gong XY Zhang YX Peng QZ Liu ZX (2019) A new cave-dwelling blind loach, *Triplophysaerythraea* sp. nov. (Cypriniformes: Nemacheilidae), from Hunan Province, China. Zoological Research 40(4): 331–336. 10.24272/j.issn.2095-8137.2019.049 31310067 PMC6680126

[B16] Kumar S Stecher G Tamura K (2016) MEGA7: Molecular evolutionary genetics analysis version 7.0 for bigger datasets. Molecular Biology and Evolution 33(7): 1870–1874. 10.1093/molbev/msw054 27004904 PMC8210823

[B17] Lan JH Yang JX Chen YR (1995) Two new species of the subfamily Nemacheilinae from Guangxi, China (Cypriniformes: Cobitidae). Dong Wu Fen Lei Xue Bao 20(3): 366–372. [In Chinese]

[B18] Lan JH Gan X Wu TJ Yang J (2013) Cave Fishes of Guangxi, China. Science Press, Beijing. [In Chinese]

[B19] Lanfear R Frandsen PB Wright AM Senfeld T Calcott B (2017) PartitionFinder 2: New methods for selecting partitioned models of evolution for molecular and morphological phylogenetic analyses. Molecular Biology and Evolution 34(3): 772–773. 10.1093/molbev/msw260 28013191

[B20] Leigh JW Bryant D (2015) POPART: full-feature software for haplotype network construction. Methods in Ecology and Evolution 6(9): 1110–1116. 10.1111/2041-210X.12410

[B21] Li WX (2004) The three new species of Cobitidae from Yunnan, China. Journal of Jishou University 25(3): 93–96. [Natural Science Edition] [In Chinese]

[B22] Li WX Zhu ZG (2000) A new species of *Triplophysa* from cave Yunnan. Journal of Yunnan University 22(5): 396–398. [Natural Science Edition] [In Chinese]

[B23] Li WX Yanf HF Chen H Tao CP Qi SQ Han NF (2008) A New Blind Underground Species of the Genus *Triplophysa* (Balitoridae) from Yunnan, China. Zoological Research 29(6): 674–678. 10.3724/SP.J.1141.2008.06674

[B24] Li J Lan JH Chen XY Du LN (2017a) Description of *Triplophysaluochengensis* sp. nov. (Teleostei: Nemacheilidae) from a karst cave in Guangxi, China. Journal of Fish Biology 91(4): 1009–1017. 10.1111/jfb.13364 28853143

[B25] Li J Li XH Lan JH Du LN (2017b) A new troglobitic loach *Triplophysatianlinensis* (Teleostei: Nemacheilidae) from Guangxi, China. Ichthyological Research 64(3): [1–6]: 295–300. 10.1007/s10228-016-0565-0

[B26] Li CQ Liu T Li R (2018) A new species of the genus Plateau loach from caves in Guizhou Province. Journal of Jishou University 39(4): 60–63. 10.13438/j.cnki.jdzk.2018.04.012 [Natural Science Edition] [In Chinese]

[B27] Li XQ Xiang XG Jabbour F Hagen O Ortiz RDC Soltis PS Soltis DE Wang W (2022) Biotic colonization of subtropical East Asian caves through time. Proceedings of the National Academy of Sciences 119(34): e2207199119. 10.1073/pnas.2207199119 PMC9407641 35969742

[B28] Liu SW Pan XF Yang JX Chen XY (2017) A new cave‐dwelling loach, *Triplophysaxichouensis* sp. nov. (TeleosteiNemacheilidae) from Yunnan, China. Journal of Fish Biology 90(3): 834–846. 10.1111/jfb.13201 28155227

[B29] Liu F Zeng ZX Gong Z (2022) Two new hypogean species of *Triplophysa* (Cypriniformes: Nemacheilidae) from the River Yangtze drainage in Guizhou, China. Journal of Vertebrate Biology 71(22062): 22062. 10.25225/jvb.22062

[B30] Lu ZM Li XJ Lü WJ Huang JQ Xu TK Huang G Qian FQ Yang P Chen SG Mao WN Zhao YH (2022) *Triplophysaxuanweiensis* sp. nov., a new blind loach species from a cave in China (Teleostei: Cypriniformes: Nemacheilidae). Zoological Research 43(2): 221–224. 10.24272/j.issn.2095-8137.2021.310 35084128 PMC8920851

[B31] Luo T Mao M-L Lan C-T Song L-X Zhao X-R Yu J Wang X-L Xiao N Zhou J-J Zhou J (2023) Four new hypogean species of the genus *Triplophysa* (Osteichthyes, Cypriniformes, Nemacheilidae) from Guizhou Province, Southwest China, based on molecular and morphological data. ZooKeys 1185: 43–81. 10.3897/zookeys.1185.105499 38074912 PMC10698870

[B32] Ma L Zhao Y Yang JX (2019) Cavefish of China. In Encyclopedia of caves (3^rd^ edn.). Academic Press, Pittsburgh, USA, 237–254. 10.1016/B978-0-12814124-3.00027-3

[B33] Microsoft Corporation (2016) Microsoft Excel. https://office.microsoft.com/excel

[B34] Nguyen LT Schmidt HA Von Haeseler A Minh BQ (2015) IQ-TREE: A fast and effective stochastic algorithm for estimating maximum-likelihood phylogenies. Molecular Biology and Evolution 32(1): 268–274. 10.1093/molbev/msu300 25371430 PMC4271533

[B35] Prokofiev AM (2010) Morphological classification of loaches (Nemacheilinae). Journal of Ichthyology 50(10): 827–913. 10.1134/S0032945210100012

[B36] Ran JC Li WX Chen HM (2006) A new species of blind loach of *Paracobitis* from Guangxi, China (Cypriniformes: Cobitidae). Guangxi Shifan Daxue Xuebao. Ziran Kexue Ban 24(3): 81–82. [In Chinese]

[B37] Ren Q Yang JX Chen XY (2012) A new species of the genus *Triplophysa* (Cypriniformes: Nemacheilidae), *Triplophysalongliensis* sp. nov., from Guizhou, China. Zootaxa 3586(1): 187–194. 10.11646/zootaxa.3586.1.17

[B38] Ronquist F Teslenko M Van Der Mark P Ayres DL Darling A Höhna S Larget B Liu L Suchard MA Huelsenbeck JP (2012) MrBayes 3.2: Efficient Bayesian phylogenetic inference and model choice across a large model space. Systematic Biology 61(3): 539–542. 10.1093/sysbio/sys029 22357727 PMC3329765

[B39] Tang L Zhao YH Zhang CG (2012) A new blind loach, *Oreonecteselongatus* sp. nov. (Cypriniformes: Balitoridae) from Guangxi, China. Environmental Biology of Fishes 93(4): 483–449. 10.1007/s10641-011-9943-7

[B40] Wang DZ Li DJ (2001) Two new species of the genus *Triplophysa* from Guizhou, China (Cypriniformes: Cobitidae). Acta Zootaxonomica Sinica 16(1): 98–101. [In hinese]

[B41] Wen H Luo T Wang Y Wang S Liu T Xiao N Zhou J (2022) Molecular phylogeny and historical biogeography of the cave fish genus *Sinocyclocheilus* (Cypriniformes: Cyprinidae) in southwest China. Integrative Zoology 17(2): 311–325. 10.1111/1749-4877.12624 34958525

[B42] Wu TJ Wei ML Lan JH Du LN (2018a) *Triplophysaanshuiensis*, a new species of blind loach from the Xijiang River, China (Teleostei, Nemacheilidae). ZooKeys 744: 67–77. 10.3897/zookeys.744.21742 PMC5904374 29670445

[B43] Wu WJ He AY Yang JX Du LN (2018b) Description of a new species of *Triplophysa* (Teleostei: Nemacheilidae) from Guizhou Province, China. Journal of Fish Biology 93(1): 88–94. 10.1111/jfb.13670 29882375

[B44] Xiao W Zhang Y Liu H (2001) Molecular systematics of Xenocyprinae (Teleostei: Cyprinidae): taxonomy, biogeography, and coevolution of a special group restricted in East Asia. Molecular Phylogenetics and Evolution 18(2): 163–173. 10.1006/mpev.2000.0879 11161753

[B45] Yang GY Yuan FX Liao YM (1986) A new blind Cobitidae fish from the subterranean water in Xiangxi, China. Huazhong Nongye Daxue Xuebao 5(3): 219–223. [In Chinese]

[B46] Yang JX Chen XY Lan JH (2004) Occurrence of two new Plateau indicator loaches of Nemacheilinae (Balitoridae) in Guangxi with Reference to Zoogeographical Significance. Zoological Research 25(2): 111–116. [In Chinese]

[B47] Yang J Wu TJ Yang JX (2012) A new cave-dwelling loach, *Triplophysamacrocephala* (Teleostei: Cypriniformes: Balitoridae), from Guangxi, China. Environmental Biology of Fishes 93(2): 169–175. 10.1007/s10641-011-9901-4

[B48] Yang HF Li WX Chen ZM (2016) A new cave species of the genus *Triplophysa* from Yunnan, China. Zoological Research 37(5): 296–300. 10.13918/j.issn.2095-8137.2016.5.296 PMC5071343 27686789

[B49] Yuan Z Wu D Wen Y Xu W Gao W Dahn HA Liu X Jin J Yu C Xiao H Che J. (2023) Historical mitochondrial genome introgression confounds species delimitation: evidence from phylogenetic inference in the *Odorranagrahami* species complex. Current Zoology 69(1): 82–90. 10.1093/cz/zoac010 36974146 PMC10039181

[B50] Zhang CG Shao GZ Wu HL Zhao YH (2020) Species Catalogue of China. Vol. 2. Animals, Vertebrates (V), Fishes. Science Press, Beijing. [In Chinese]

[B51] Zhao YH Gozlan RE Zhang CG (2011) Out of sight out of mind: Current knowledge of Chinese cave fishes. Journal of Fish Biology 79(6): 1545–1562. 10.1111/j.1095-8649.2011.03066.x 22136239

[B52] Zheng LP Du LN Chen XY Yang JX (2009) A new species of Genus *Triplophysa* (Nemacheilinae: Balitoridae), *Triplophysalongipectoralis* sp. nov., from Guangxi, China. Environmental Biology of Fishes 85(3): 221–227. 10.1007/s10641-009-9485-4

[B53] Zheng LP Yang JX Chen XY (2012) A new species of *Triplophysa* (Nemacheilidae: Cypriniformes), from Guangxi, southern China. Journal of Fish Biology 80(4): 831–841. 10.1111/j.1095-8649.2012.03227.x 22471802

[B54] Zhou QY Chen PY (1993) Lithofacies change and palaeogeographical evolution during Late Cenozoic in Guizhou and its vicinity. Geology of Guizhou 10(3): 201–207.

[B55] Zhu SQ (1989) The loaches of the subfamily Nemacheilinae in China (Cypriniformes: Cobitidae). Jiangsu Science and Technology Publishing House, Nanjing, 150 pp. [In Chinese]

[B56] Zhu SQ Cao WX (1988) Descriptions of two new species and a new subspecies of Noemacheilinae from Yunnan Province (Cypriniformes: Cobitidae). Dong Wu Fen Lei Xue Bao (1): 95–100. [In Chinese]

