# Supplementary material for: ﻿Two new hypogean species of the genus Triplophysa (Osteichthyes, Cypriniformes, Nemacheilidae) from Guizhou Province, Southwest China, with underestimated diversity
Source: Zookeys. 2024 Oct 9;1214:237–64. doi: 10.3897/zookeys.1214.122439 (PMC11484637; doi:10.3897/zookeys.1214.122439)
Supplement: Supplementary material 1 — Morphological characters and measurement data of the new species Triplophysaziyunensis sp. nov., Triplophysayaluwang sp. nov., T.wudangensis, T.rosa, T.qingzhenensis, and T.guizhouensis [file zookeys-1214-237_article-122439__-s001.docx]

**Table S1** Morphological characters and measurement data of the new species *Triplophysa ziyunensis* sp. nov., *Triplophysa yaluwang* sp. nov., *T. wudangensis*, *T. rosa*, *T. qingzhenensis*, and *T. guizhouensis*. *Designates the holotype.

| Species | Voucher number (*Holotype) | Dorsal-fin rays | Pectoral-fin rays | Pelvic-fin rays | Anal-fin rays |
| --- | --- | --- | --- | --- | --- |
| Triplophysa yaluwang sp. nov. | **GZNU20240118001*** | iii,7 | i,9 | i,5 | iii,5 |
| Triplophysa yaluwang sp. nov. | GZNU20240118002 | iii,7 | i,9 | i,5 | iii,5 |
| Triplophysa yaluwang sp. nov. | GZNU20240118003 | iii,7 | i,9 | i,5 | iii,5 |
| Triplophysa yaluwang sp. nov. | GZNU20240118004 | iii,7 | i,9 | i,5 | iii,5 |
| Triplophysa yaluwang sp. nov. | GZNU20240118005 | iii,7 | i,9 | i,5 | iii,5 |
| Triplophysa ziyunensis sp. nov. | GZNU20230226008 | iii,8 | i,10 | i,6 | iii,5 |
| Triplophysa ziyunensis sp. nov. | GZNU20230226009 | iii,8 | i,10 | i,6 | iii,5 |
| Triplophysa ziyunensis sp. nov. | GZNU20230226010 | iii,8 | i,10 | i,6 | iii,5 |
| Triplophysa ziyunensis sp. nov. | **GZNU20230529001*** | iii,8 | i,10 | i,6 | iii,5 |
| Triplophysa ziyunensis sp. nov. | GZNU20230529002 | iii,8 | i,10 | i,6 | iii,5 |
| *Triplophysa rosa* | GZNU20230216070 | iii, 9 | i, 12 | i, 7 | iii, 6 |
| *Triplophysa rosa* | GZNU20230216071 | iii, 9 | i, 12 | i, 7 | iii, 6 |
| *Triplophysa rosa* | GZNU20230216072 | iii, 9 | i, 12 | i, 7 | iii, 6 |
| *Triplophysa rosa* | GZNU20230216073 | iii, 9 | i, 12 | i, 7 | iii, 6 |
| *Triplophysa rosa* | GZNU20230216074 | iii, 9 | i, 12 | i, 7 | iii, 6 |
| *Triplophysa rosa* | GZNU20230216075 | iii, 9 | i, 12 | i, 7 | iii, 6 |
| *Triplophysa rosa* | GZNU20230216076 | iii, 9 | i, 12 | i, 7 | iii, 6 |
| *Triplophysa rosa* | GZNU20230216077 | iii, 9 | i, 12 | i, 7 | iii, 6 |
| *Triplophysa rosa* | GZNU20230216078 | iii, 9 | i, 12 | i, 7 | iii, 6 |
| *Triplophysa wudangensis* | GZNU2023002160 | iii, 7 | i, 8 | i, 5 | iii, 5 |
| *Triplophysa wudangensis* | GZNU20240122001 | iii, 7 | i, 8 | i, 5 | iii, 5 |
| *Triplophysa wudangensis* | GZNU20240122002 | iii, 7 | i, 8 | i, 5 | iii, 5 |
| *Triplophysa qingzhenensis* | GZNU20240122003 | iii, 7 | 10 | i, 6 | iii, 6 |
| *Triplophysa qingzhenensis* | GZNU20240122004 | iii, 7 | 11 | i, 5 | iii, 6 |
| *Triplophysa qingzhenensis* | GZNU20240122005 | iii, 7 | 11 | i, 6 | iii, 6 |
| *Triplophysa qingzhenensis* | GZNU20240122006 | iii, 7 | 11 | i, 6 | iii, 5 |
| *Triplophysa qingzhenensis* | GZNU20240122007 | iii, 7 | 10 | i, 5 | iii, 6 |
| *Triplophysa qingzhenensis* | GZNU20240122008 | iii, 8 | 11 | i, 6 | iii, 6 |
| *Triplophysa qingzhenensis* | GZNU20240122009 | iii, 7 | 11 | i, 6 | iii, 5 |
| *Triplophysa qingzhenensis* | GZNU20240122010 | iii, 7 | 11 | i, 6 | iii, 6 |
| *Triplophysa guizhouensis* | GZNU20230722001 | iii, 8 | i,9 | i, 6 | iii, 6 |
| *Triplophysa guizhouensis* | GZNU20230722002 | iii, 8 | i,9 | i, 6 | iii, 6 |
| *Triplophysa guizhouensis* | GZNU20230722003 | iii, 8 | i,9 | i, 6 | iii, 6 |
| *Triplophysa guizhouensis* | GZNU20230722004 | iii, 8 | i,9 | i, 6 | iii, 6 |
| *Triplophysa guizhouensis* | GZNU20230722005 | iii, 8 | i,9 | i, 6 | iii, 6 |
| *Triplophysa guizhouensis* | GZNU20230722006 | iii, 8 | i,9 | i, 6 | iii, 6 |
| *Triplophysa guizhouensis* | GZNU20230722007 | iii, 8 | i,9 | i, 6 | iii, 6 |
| *Triplophysa longliensis* | KIZ2010002988* | - | - | - | - |
| *Triplophysa sanduensis* | SWU2017061305* | - | - | - | - |

……continued on the next page

Table S1. (Continued)

| Species | Caudal-fin rays | Total length | Standard length | Head length | Head depth | Head width | Snout length |
| --- | --- | --- | --- | --- | --- | --- | --- |
| *Triplophysa yaluwang* **sp. nov.** | 14 | 87.6 | 73.9 | 16.8 | 8.4 | 10.9 | 8.7 |
| *Triplophysa yaluwang* **sp. nov.** | 14 | 99.4 | 83.9 | 19.7 | 9.8 | 12.4 | 9.3 |
| *Triplophysa yaluwang* **sp. nov.** | 14 | 66.7 | 54.1 | 13.4 | 6.1 | 8.1 | 5.7 |
| *Triplophysa yaluwang* **sp. nov.** | 14 | 66.5 | 55.1 | 13.4 | 6.1 | 7.0 | 6.1 |
| *Triplophysa yaluwang* **sp. nov.** | 14 | 66.9 | 54.7 | 13.4 | 6.5 | 6.9 | 0.0 |
| *Triplophysa ziyunensis* **sp. nov.** | 16 | 78.6 | 63.3 | 17.4 | 8.6 | 10.5 | 8.6 |
| *Triplophysa ziyunensis* **sp. nov.** | 16 | 115.8 | 95.3 | 25.5 | 11.9 | 15.0 | 11.9 |
| *Triplophysa ziyunensis* **sp. nov.** | 16 | 120.0 | 100.1 | 26.2 | 11.6 | 16.1 | 12.0 |
| *Triplophysa ziyunensis* **sp. nov.** | 16 | 105.1 | 86.7 | 22.7 | 11.1 | 12.5 | 10.8 |
| *Triplophysa ziyunensis* **sp. nov.** | 16 | 99.8 | 80.4 | 20.6 | 9.1 | 10.5 | 8.9 |
| *Triplophysa rosa* | 14 | 130.8 | 104.3 | 28.6 | 16.1 | 17.2 | - |
| *Triplophysa rosa* | 14 | 120.2 | 96.6 | 26.6 | 13.6 | 11.7 | - |
| *Triplophysa rosa* | 14 | 111.0 | 90.3 | 25.2 | 12.5 | 14.4 | - |
| *Triplophysa rosa* | 14 | 89.9 | 72.4 | 20.6 | 9.8 | 11.2 | - |
| *Triplophysa rosa* | 14 | 87.8 | 70.8 | 21.0 | 9.7 | 11.8 | 10.4 |
| *Triplophysa rosa* | 14 | 80.3 | 64.7 | 19.2 | 9.0 | 11.4 | - |
| *Triplophysa rosa* | 14 | 75.5 | 61.7 | 17.3 | 8.5 | 10.4 | 9.0 |
| *Triplophysa rosa* | 14 | 68.2 | 55.6 | 16.2 | 7.7 | 9.7 | 8.1 |
| *Triplophysa rosa* | 14 | 62.3 | 49.8 | 14.7 | 6.8 | 8.8 | 7.3 |
| *Triplophysa wudangensis* | 14 | 73.4 | 59.8 | 11.5 | 6.6 | 8.2 | 6.2 |
| *Triplophysa wudangensis* | 14 | 79.3 | 64.6 | 12.5 | 7.1 | 8.9 | 6.7 |
| *Triplophysa wudangensis* | 14 | 85.9 | 66.8 | 12.9 | 7.4 | 9.2 | 7.0 |
| *Triplophysa qingzhenensis* | 14 | 121.6 | 102.8 | 23.4 | 12.2 | 14.6 | 11.6 |
| *Triplophysa qingzhenensis* | 14 | 118.3 | 97.9 | 22.7 | 11.3 | 13.8 | 11.5 |
| *Triplophysa qingzhenensis* | 14 | 117.2 | 98.4 | 22.6 | 12.1 | 14.5 | 11.8 |
| *Triplophysa qingzhenensis* | 14 | 111.1 | 91.5 | 21.5 | 11.7 | 13.7 | 10.8 |
| *Triplophysa qingzhenensis* | 14 | 123.3 | 103.0 | 24.4 | 12.9 | 15.8 | 12.3 |
| *Triplophysa qingzhenensis* | 14 | 107.6 | 90.4 | 22.5 | 12.8 | 14.5 | 11.6 |
| *Triplophysa qingzhenensis* | 14 | 93.1 | 77.7 | 16.5 | 8.6 | 10.7 | 8.4 |
| *Triplophysa qingzhenensis* | 14 | 84.6 | 72.2 | 15.8 | 8.8 | 10.2 | 7.9 |
| *Triplophysa guizhouensis* | 14 | 81.0 | 67.2 | 6.2 | 7.4 | 10.7 | 6.6 |
| *Triplophysa guizhouensis* | 14 | 81.1 | 67.5 | 15.4 | 7.4 | 9.9 | 6.4 |
| *Triplophysa guizhouensis* | 14 | 69.2 | 56.7 | 13.3 | 6.4 | 9.1 | 5.4 |
| *Triplophysa guizhouensis* | 14 | 88.1 | 71.4 | 15.9 | 8.3 | 10.9 | 6.9 |
| *Triplophysa guizhouensis* | 14 | 67.2 | 55.9 | 14.5 | 7.4 | 9.4 | 6.6 |
| *Triplophysa guizhouensis* | 14 | 54.9 | 45.8 | 10.9 | 5.8 | 7.1 | 5.2 |
| *Triplophysa guizhouensis* | 14 | 86.9 | 72.8 | 16.7 | 7.8 | 8.8 | 7.4 |
| *Triplophysa longliensis* | - | 99.4 | 81.6 | 18.2 | 8.5 | 9.9 | 7.2 |
| *Triplophysa sanduensis* | - | 80.5 | 67.9 | 15.4 | 8.0 | 8.2 | 7.7 |

……continued on the next page

Table S1. (Continued)

| Species | Eye diameter | Interorbital  distance | Body depth | Body width | Maxillary  barbel length | Outrostral  barbel length | Inrostral  barbel length |
| --- | --- | --- | --- | --- | --- | --- | --- |
| *Triplophysa yaluwang* **sp. nov.** | 0.8 | 4.3 | 10.2 | 8.3 | 5.6 | 7.3 | 4.5 |
| *Triplophysa yaluwang* **sp. nov.** | 1.1 | 5.0 | 13.5 | 9.1 | 6.8 | 8.1 | 4.4 |
| *Triplophysa yaluwang* **sp. nov.** | 0.8 | 3.5 | 7.9 | 8.2 | 4.6 | 5.5 | 2.2 |
| *Triplophysa yaluwang* **sp. nov.** | 0.6 | 3.3 | 6.5 | 8.1 | 4.8 | 5.7 | 3.1 |
| *Triplophysa yaluwang* **sp. nov.** | 0.0 | 0.0 | 7.4 | 8.8 | 3.0 | 5.3 | 3.6 |
| *Triplophysa ziyunensis* **sp. nov.** | 0.4 | 4.5 | 9.4 | 7.6 | 6.0 | 10.1 | 4.6 |
| *Triplophysa ziyunensis* **sp. nov.** | 1.1 | 6.0 | 13.3 | 12.9 | 9.1 | 13.2 | 7.0 |
| *Triplophysa ziyunensis* **sp. nov.** | 1.2 | 6.5 | 14.7 | 13.2 | 9.5 | 14.5 | 6.1 |
| *Triplophysa ziyunensis* **sp. nov.** | 0.7 | 5.2 | 13.7 | 10.7 | 9.3 | 12.2 | 5.6 |
| *Triplophysa ziyunensis* **sp. nov.** | 1.0 | 4.6 | 10.1 | 8.7 | 8.6 | 10.6 | 5.4 |
| *Triplophysa rosa* | 0.0 | - | 17.2 | 14.7 | 10.3 | 10.6 | 6.5 |
| *Triplophysa rosa* | 0.0 | - | 15.7 | 11.4 | 10.0 | 9.8 | 5.3 |
| *Triplophysa rosa* | 0.0 | - | 12.4 | 10.2 | 9.5 | 10.1 | 5.4 |
| *Triplophysa rosa* | 0.0 | - | 8.6 | 6.2 | 7.0 | 8.3 | 4.4 |
| *Triplophysa rosa* | 0.0 | 6.8 | 8.5 | 6.5 | 7.0 | 8.7 | 4.1 |
| *Triplophysa rosa* | 0.0 | - | 8.3 | 6.5 | 7.4 | 7.1 | 3.7 |
| *Triplophysa rosa* | 0.0 | 6.0 | 7.9 | 5.8 | 6.1 | 6.5 | 3.2 |
| *Triplophysa rosa* | 0.0 | 5.5 | 7.6 | 5.4 | 5.1 | 7.5 | 4.3 |
| *Triplophysa rosa* | 0.4 | 4.7 | 5.9 | 4.3 | 5.3 | 6.6 | 3.5 |
| *Triplophysa wudangensis* | 0.6 | 4.0 | 6.6 | 4.8 | 5.2 | 7.2 | 3.1 |
| *Triplophysa wudangensis* | 0.6 | 4.4 | 7.1 | 5.2 | 5.7 | 7.7 | 3.4 |
| *Triplophysa wudangensis* | 0.7 | 4.5 | 7.3 | 5.4 | 5.9 | 8.0 | 3.5 |
| *Triplophysa qingzhenensis* | 0.2 | 7.2 | 16.9 | 15.6 | 7.9 | 7.8 | 4.3 |
| *Triplophysa qingzhenensis* | 0.2 | 7.1 | 14.4 | 13.5 | 9.1 | 10.1 | 5.3 |
| *Triplophysa qingzhenensis* | 0.2 | 7.7 | 15.9 | 14.7 | 7.4 | 10.0 | 4.8 |
| *Triplophysa qingzhenensis* | 0.2 | 7.2 | 14.9 | 13.3 | 7.7 | 9.3 | 5.1 |
| *Triplophysa qingzhenensis* | 0.3 | 7.7 | 17.0 | 15.2 | 7.4 | 8.8 | 5.4 |
| *Triplophysa qingzhenensis* | 0.2 | 7.1 | 15.0 | 12.8 | 9.5 | 10.6 | 4.8 |
| *Triplophysa qingzhenensis* | 0.2 | 5.5 | 12.5 | 10.6 | 6.5 | 7.9 | 3.9 |
| *Triplophysa qingzhenensis* | 0.3 | 5.1 | 11.7 | 10.8 | 5.5 | 6.7 | 3.8 |
| *Triplophysa guizhouensis* | 1.7 | 4.1 | 10.1 | 7.6 | 5.0 | 7.1 | 2.9 |
| *Triplophysa guizhouensis* | 1.8 | 3.1 | 8.4 | 6.6 | 5.8 | 5.7 | 3.0 |
| *Triplophysa guizhouensis* | 2.0 | 2.1 | 7.6 | 5.8 | 4.6 | 6.1 | 3.3 |
| *Triplophysa guizhouensis* | 2.3 | 4.3 | 11.2 | 9.8 | 5.4 | 5.8 | 3.4 |
| *Triplophysa guizhouensis* | 1.8 | 3.3 | 7.6 | 7.0 | 5.2 | 6.7 | 3.9 |
| *Triplophysa guizhouensis* | 2.0 | 2.3 | 6.3 | 5.9 | 3.9 | 4.6 | 2.8 |
| *Triplophysa guizhouensis* | 2.0 | 3.9 | 10.1 | 8.1 | 6.4 | 7.7 | 3.9 |
| *Triplophysa longliensis* | 2.1 | 5.9 | 10.9 | 9.7 | 5.9 | 6.5 | 3.4 |
| *Triplophysa sanduensis* | 1.8 | 5.8 | 10.9 | 7.3 | 5.5 | 6.3 | 3.4 |

……continued on the next page

Table S1. (Continued)

| Species | Dorsal-fin length | Dorsal-fin  base length | Pectoral-fin length | Anal-fin length | Pelvic-fin length | Caudal peduncle  length | Caudal  peduncle depth |
| --- | --- | --- | --- | --- | --- | --- | --- |
| *Triplophysa yaluwang* **sp. nov.** | 14.9 | 9.1 | 14.3 | 11.7 | 11.5 | 13.0 | 5.3 |
| *Triplophysa yaluwang* **sp. nov.** | 16.1 | 10.3 | 16.8 | 13.5 | 13.3 | 16.5 | 6.6 |
| *Triplophysa yaluwang* **sp. nov.** | 11.1 | 7.2 | 11.3 | 9.6 | 9.2 | 10.7 | 4.2 |
| *Triplophysa yaluwang* **sp. nov.** | 11.8 | 5.9 | 11.8 | 9.4 | 9.3 | 9.8 | 4.0 |
| *Triplophysa yaluwang* **sp. nov.** | 11.3 | 6.7 | 13.9 | 9.2 | 9.0 | 10.0 | 3.8 |
| *Triplophysa ziyunensis* **sp. nov.** | 15.0 | 9.6 | 13.7 | 11.9 | 11.6 | 9.8 | 4.8 |
| *Triplophysa ziyunensis* **sp. nov.** | 22.6 | 13.3 | 21.7 | 17.5 | 19.3 | 13.5 | 6.5 |
| *Triplophysa ziyunensis* **sp. nov.** | 22.1 | 13.4 | 20.2 | 18.2 | 15.9 | 16.9 | 6.7 |
| *Triplophysa ziyunensis* **sp. nov.** | 17.5 | 11.7 | 18.3 | 14.1 | 15.1 | 16.2 | 5.9 |
| *Triplophysa ziyunensis* **sp. nov.** | 19.0 | 11.1 | 18.7 | 16.2 | 14.8 | 11.6 | 5.3 |
| *Triplophysa rosa* | 26.9 | 15.8 | 35.9 | 25.3 | 24.4 | 16.8 | 9.3 |
| *Triplophysa rosa* | 22.1 | 13.9 | 22.9 | 19.5 | 19.3 | 17.7 | 8.9 |
| *Triplophysa rosa* | 20.8 | 14.4 | 23.4 | 17.1 | 17.8 | 14.0 | 6.3 |
| *Triplophysa rosa* | 17.7 | 10.3 | 22.4 | 17.1 | 15.2 | 11.4 | 4.8 |
| *Triplophysa rosa* | 17.7 | 9.8 | 19.4 | 14.3 | 13.7 | 11.0 | 4.7 |
| *Triplophysa rosa* | 15.8 | 8.4 | 19.8 | 15.3 | 13.0 | 9.4 | 4.5 |
| *Triplophysa rosa* | 15.7 | 8.8 | 15.3 | 12.9 | 12.8 | 9.2 | 4.4 |
| *Triplophysa rosa* | 14.0 | 7.4 | 15.2 | 10.9 | 10.7 | 8.5 | 4.0 |
| *Triplophysa rosa* | 13.4 | 7.5 | 14.4 | 9.5 | 8.9 | 8.7 | 3.1 |
| *Triplophysa wudangensis* | 28.3 | 7.3 | 12.0 | 9.8 | 9.2 | 11.8 | 3.9 |
| *Triplophysa wudangensis* | 30.6 | 7.9 | 12.9 | 10.6 | 9.9 | 12.7 | 4.2 |
| *Triplophysa wudangensis* | 31.7 | 8.2 | 13.4 | 10.9 | 10.2 | 13.1 | 4.4 |
| *Triplophysa qingzhenensis* | 18.4 | 9.6 | 17.3 | 14.3 | 13.5 | 14.8 | 9.4 |
| *Triplophysa qingzhenensis* | 12.3 | 8.3 | 21.7 | 16.1 | 16.8 | 17.4 | 7.5 |
| *Triplophysa qingzhenensis* | 18.6 | 9.4 | 18.6 | 14.8 | 14.2 | 17.7 | 8.3 |
| *Triplophysa qingzhenensis* | 19.3 | 7.9 | 19.3 | 15.5 | 15.2 | 17.8 | 8.0 |
| *Triplophysa qingzhenensis* | 20.1 | 8.9 | 19.8 | 15.1 | 16.2 | 18.2 | 8.7 |
| *Triplophysa qingzhenensis* | 17.5 | 8.7 | 17.5 | 14.0 | 13.8 | 16.7 | 8.2 |
| *Triplophysa qingzhenensis* | 15.0 | 9.2 | 14.3 | 11.6 | 11.5 | 12.9 | 6.8 |
| *Triplophysa qingzhenensis* | 13.1 | 8.4 | 14.4 | 10.2 | 10.8 | 11.9 | 6.5 |
| *Triplophysa guizhouensis* | 12.6 | 8.9 | 10.4 | 10.4 | 12.3 | 11.0 | 5.0 |
| *Triplophysa guizhouensis* | 13.8 | 8.3 | 11.2 | 11.2 | 12.6 | 10.3 | 5.4 |
| *Triplophysa guizhouensis* | 11.6 | 7.8 | 9.7 | 10.3 | 10.9 | 8.1 | 4.2 |
| *Triplophysa guizhouensis* | 14.4 | 9.1 | 12.2 | 11.5 | 13.0 | 12.4 | 5.9 |
| *Triplophysa guizhouensis* | 12.8 | 7.7 | 9.5 | 9.7 | 11.4 | 8.2 | 3.8 |
| *Triplophysa guizhouensis* | 9.0 | 5.6 | 7.4 | 8.0 | 9.0 | 6.6 | 3.5 |
| *Triplophysa guizhouensis* | 13.8 | 9.5 | 11.6 | 10.3 | 14.7 | 12.8 | 5.7 |
| *Triplophysa longliensis* | 18.2 | 9.2 | 16.1 | 14.6 | 13.8 | 14.0 | 5.1 |
| *Triplophysa sanduensis* | 12.2 | 8.8 | 11.8 | 9.6 | 9.7 | 11.9 | 7.3 |
