# Supplementary material for: ﻿Two new hypogean species of the genus Triplophysa (Osteichthyes, Cypriniformes, Nemacheilidae) from Guizhou Province, Southwest China, with underestimated diversity
Source: Zookeys. 2024 Oct 9;1214:237–64. doi: 10.3897/zookeys.1214.122439 (PMC11484637; doi:10.3897/zookeys.1214.122439)
Supplement: Supplementary material 2 — Results and percentage of variance explained by principal component analysis [file zookeys-1214-237_article-122439__-s002.docx]

**Table S2** Results and percentage of variance explained by principal component analysis.

|  | *Triplophysa ziyunensis* **sp. nov.** | | | | *Triplophysa yaluwang* **sp. nov.** | | | |
| --- | --- | --- | --- | --- | --- | --- | --- | --- |
|  | PC 1 | PC 2 | PC 3 | PC 4 | PC 1 | PC 2 | PC 3 | PC 4 |
| Standard length | -0.647 | 0.501 | 0.32 | 0.088 | 0.483 | -0.269 | 0.52 | 0.216 |
| Head length | 0.769 | 0.455 | 0.347 | -0.05 | -0.166 | 0.334 | 0.471 | 0.487 |
| Head depth | 0.547 | 0.692 | 0.079 | -0.152 | 0.425 | 0.343 | 0.683 | -0.09 |
| Head width | 0.44 | 0.33 | 0.536 | -0.455 | 0.566 | 0.445 | -0.022 | -0.247 |
| Snout length | -0.472 | -0.222 | 0.77 | -0.279 | 0.759 | -0.122 | -0.01 | 0.558 |
| Eye diameter | -0.195 | -0.719 | 0.329 | 0.464 | 0.769 | 0.213 | -0.381 | -0.027 |
| Interorbital distance | -0.5 | -0.197 | 0.68 | -0.436 | 0.514 | -0.489 | -0.133 | 0.578 |
| Body depth | -0.453 | 0.801 | 0.136 | 0.297 | 0.342 | -0.672 | 0.26 | -0.266 |
| Body width | -0.533 | 0.722 | 0.184 | 0.315 | -0.653 | 0.222 | 0.243 | -0.032 |
| Maxillary barbel length | 0.723 | -0.014 | 0.12 | 0.264 | 0.747 | 0.154 | 0.071 | 0.526 |
| Outrostral barbel length | 0.388 | -0.468 | 0.653 | 0.323 | 0.429 | 0.414 | 0.502 | -0.132 |
| Inrostral barbel length | 0.601 | -0.092 | 0.605 | 0.275 | 0.086 | 0.545 | 0.737 | -0.135 |
| Dorsal-fin length | 0.013 | -0.834 | -0.313 | -0.183 | -0.221 | 0.744 | -0.039 | 0.472 |
| Dorsal-fin base length | 0.801 | -0.197 | 0.07 | 0.235 | 0.684 | 0.02 | 0.207 | -0.49 |
| Pectoral-fin length | 0.888 | 0.206 | -0.155 | -0.2 | -0.882 | 0.028 | 0.419 | 0.111 |
| Anal-fin length | 0.922 | 0.122 | -0.175 | -0.048 | -0.226 | 0.739 | -0.208 | 0.246 |
| Pelvic-fin length | 0.881 | 0.263 | -0.064 | 0.036 | 0.503 | 0.631 | -0.257 | -0.208 |
| Caudal peduncle length | -0.615 | -0.244 | -0.179 | 0.01 | -0.476 | -0.64 | 0.319 | 0.207 |
| Caudal peduncle depth | -0.54 | 0.753 | -0.145 | 0.09 | 0.395 | -0.703 | 0.275 | -0.009 |
| Eigenvalues | 3.018 | 1.858 | 3.798 | 0.594 | 4.078 | 1.937 | 3.657 | 1.765 |
| Percentage of total variance | 38.232 | 23.839 | 14.495 | 6.850 | 28.771 | 22.086 | 13.448 | 10.559 |
| Cumulative percentage | 38.232 | 62.071 | 76.566 | 83.416 | 28.771 | 50.857 | 64.304 | 74.863 |
