# Supplementary material for: ﻿Two new hypogean species of the genus Triplophysa (Osteichthyes, Cypriniformes, Nemacheilidae) from Guizhou Province, Southwest China, with underestimated diversity
Source: Zookeys. 2024 Oct 9;1214:237–64. doi: 10.3897/zookeys.1214.122439 (PMC11484637; doi:10.3897/zookeys.1214.122439)
Supplement: Supplementary material 3 — Specimens examined in this work [file zookeys-1214-237_article-122439__-s003.docx]

**Supplementary file 3. Specimens examined in this work.**

***Triplophysa anlongensis*** (*n* = 7): China: Guizhou: Anlong County: Xinglong Town: NaNao Village: GZNU20230226002, GZNU20230105001, GZNU20230215025–215029, GZNU20230216042–216044, GZNU20230226001–226003.

***Triplophysa cehengensis*** (*n* = 3): China: Guizhou: Ceheng County: Rongdu Town: Longjing Village: GZNU20230214010–214012.

***Triplophysa baotianensis*** (*n* = 5): China: Guizhou: Panzhou City: Baotian Town (type locality): GZNU 20180421001–421005.

***Triplophysa erythraea*** (*n* = 2): China: Hunan: Huayuan County: Dalong Cave (type locality): GZNU20230216052 and GZNU20230216053.

***Triplophysa guizhouensis*** (*n* = 7): China: Guizhou: Huisui County: Baijin Town (type locality): GZNU 20220715001–0715007.

***Triplophysa huapingensis*** (*n* = 7): China: Guangxi: Leye County: Huaping Township (type locality): GZNU 20221209012–1209019.

***Triplophysa langpingensis*** (*n* = 2): China: Guangxi: Leye County: Huaping Township (type locality): GZNU 20221209001–1209002.

***Triplophysa macrocephala*** (*n* = 8): China: Guangxi: Nandan County: Lihu Township (type locality): GZNU 20221209003–1209010.

***Triplophysa nasobarbatula*** (*n* = 8): China: Guizhou: Libo County: Dongtang Township (type locality): GZNU 20170731003–0731010.

***Triplophysa nandanensis*** (*n* = 2): China: Guangxi: Nandan County: Liuzhai Town (type locality): GZNU 20221209020–1209021.

***Triplophysa panzhouensis*** (*n* = 12): China: Guizhou: Ceheng County: Rongdu Town: Rongbei Village: GZNU20230226002, GZNU20230105001, GZNU20230215025–215029, GZNU20230216042–216044, GZNU20230226001–226003.

***Triplophysa qingzhenensis*** (*n* = 8): China: Guizhou: Guiyang City: Qingzhen County (type locality): GZNU20220830001–0830008.

***Triplophysa qini*** (*n* = 5): China: Chongqing: Wulong District: Fengdu County: Dudu Village (type locality): GZNU20230216045–0216045.

***Triplophysa qiubeiensis*** (*n* = 2): China: Yunan: Qiubei County: Nijiao Town (type locality): GZNU 20221209022–1209023.

***Triplophysa rosa*** (*n* = 9): China: Chongqing: Wulong County: Huolu Town: GZNU20230216070–0216078.

***Triplophysa rongduensis*** (*n* = 6): China: Guizhou: Ceheng County: Rongdu Town: Rongbei Village: GZNU20230106001, GZNU20230214001–214005.

***Triplophysa tianeensis*** (*n* = 7): China: Guangxi: Tian'e County: Bala Township (type locality): GZNU 20221209011–1209017.

***Triplophysa wudangensis*** (*n* = 4): China: Guizhou: Guiyang City: Wudang District (type locality): IHB 201908090001–090004.

***Triplophysa zhenfengensis*** (*n* = 5): China: Guizhou: Xingren City: Xinlongchang Town: GZNU20180419002, GZNU20190521001–052104.
